# A pioneer of acrylic painting: new insights into Carmen Herrera’s studio practice

**DOI:** 10.1186/s40494-021-00603-3

**Published:** 2021-10-14

**Authors:** Federica Pozzi, Elena Basso, Silvia A. Centeno, Isabelle Duvernois, Julie Arslanoglu

**Affiliations:** 1 Department of Scientific Research, The Metropolitan Museum of Art, 1000 Fifth Avenue, New York, NY 10028 USA; 2 Department of Paintings Conservation, The Metropolitan Museum of Art, 1000 Fifth Avenue, New York, NY 10028 USA; 3 Center for Conservation and Restoration of Cultural Heritage “La Venaria Reale”, Via XX Settembre 18, 10078 Venaria Reale (Torino), Italy

**Keywords:** Carmen Herrera, Contemporary art, Abstract art, Latin American art, Painting, Studio practice, Binding media, Acrylics

## Abstract

Carmen Herrera, born on May 30th, 1915, is a Cuban American abstract minimalist artist, whose first solo show was held at the Whitney Museum of American Art, New York, in 2016–2017. On this occasion, a scientific study of five paintings from the artist’s time in Paris (1948–1953) revealed her pioneering use of solvent-based acrylic paints in post-war Europe. This article presents a second phase of research into Herrera’s work aiming to shed light on her studio practice and ascertain the possible presence of other early acrylic paints in her pre-1963 artistic production. A selection of four paintings, namely *Iberia #25* (1948), *Iberic* (1949), *Flights of Colors #16* (1949), and *Early Dynasty* (1953), was subjected to an analytical campaign that relied on both non-invasive and micro-invasive techniques. Results confirmed the use of both oil and solvent-based acrylic paints, supporting our primary research and uncovering the first-known occurrence of acrylic binders in Herrera’s *Iberia #25*. In all cases, the ground layer consists of a mixture of titanium white in its tetragonal form of rutile, anhydrite, and talc, while the color palette was found to be based on both traditional and modern pigments. In most areas, several paint layers appeared to be overlaid on top of one another, revealing a creative process that developed through subsequent compositions. Remnants of earlier paint layers, which appeared to have been scraped off before new ones were applied, were observed directly above the ground in some of the samples examined. In addition, *Iberia #25* and *Iberic*, with analogous geometric and chromatic schemes, underwent technical imaging, which exposed pencil lines and notes underneath the paint layers likely indicative of the intended design and polychromy. Besides corroborating a major alteration in the current scholarship on the availability and use of acrylic-based artists’ paints in post-war Europe, this research provides new insights into Herrera’s materials, techniques, and studio practice. In addition, the results of this scientific study assisted the development of a suitable treatment plan for *Iberic* in preparation for its display in The Metropolitan Museum of Art’s galleries as part of the museum’s 150th anniversary exhibition *Making The Met, 1870–2020*.

**Supplementary Information:**

The online version contains supplementary material available at 10.1186/s40494-021-00603-3.

## Introduction

Currently 106 years old, minimalist visual artist Carmen Herrera was born in Havana, Cuba. A master of geometric abstraction, her painting compositions are often dominated by crisp lines and contrasting chromatic planes, likely influenced by her academic studies in architecture and singularly focused on the interactions between space and color. Distinctive traits of Herrera’s painting style are a drive for formal simplicity, an unafraid exploration of the concepts of balance and asymmetry, and a striking sense of color. She stated of her own work, “*My quest is for the simplest of pictorial resolutions*”. Having sold her first painting at the age of 89, Herrera achieved international success late in life and only recently began to receive proper recognition by the public and the press. Questioned by journalists about her experience as a woman artist in the post-war years, she explained that she faced some obstacles early in her career “*because everything was controlled by men, not just art*”.

Herrera’s first solo show, entitled *Carmen Herrera: Lines of Sight* [1], was held at the Whitney Museum of American Art, New York, in 2016–2017. Featuring more than fifty works, the exhibition focused on a thirty-year period that started with the artist’s arrival in Paris in 1948, where she worked for about six years before returning to New York City. During installation, Herrera indicated the binding media for all of the paintings in the show as being acrylics, even though the earliest works dated from the late 1940s to the early 1950s, when she was living in Europe and acrylics were not yet known to be commercially available there.

Dana Miller, the exhibition’s curator, had numerous exchanges with Herrera between 2013 and 2016, in which the artist insisted that she began using acrylic paints during her time in Paris. Questioned multiple times about this topic, Herrera was always resolute and specific about using acrylics, even when countered with the prevailing belief that acrylic-based artists’ paints were not available in Europe in the 1940–50s; she even recalled buying them in an art supply store near her studio in the French capital. According to Miller, “*Herrera cares deeply about her materials and she would not be mistaken about this. If she didn't know or didn't recall, she would have said so*”^1^. In fact, Herrera’s discussing of her own use of early acrylics imported from Germany while she lived in Paris had already been recorded in a 2005 interview with Estrellita Brodsky, in which the artist stated: “*Above all, for the type of painting that I do, it was a blessing from God*”^2^. Herrera’s certain and consistent recollection, Miller explained, is the reason why a decision was made to keep the term “acrylic” in the media assignments for the Whitney Museum exhibition before her statements could be verified through chemical analysis of samples from her paintings^1^.

Herrera’s revelations prompted a first scientific investigation of binding media in five paintings from the artist’s time in Paris (1948–1953), carried out by some of the authors of this article and previously published in the *Journal of Cultural Heritage* [2]. Unfortunately for the research team, none of the paint tubes from Herrera’s French studio were existent any longer. Interestingly, the study revealed a complex progression of organic binders in the works examined: mixtures of modified oils, detected in paintings dated to 1948 and 1949, were found to have been gradually replaced or combined with other binding media, such as solvent-based acrylics—mostly *n*-butyl methacrylate—polyvinyl acetate, and oil-based alkyds (based on *ortho*-phthalic acid), in works painted during the following three years. Results corroborated Herrera’s memories and highlighted her pioneering use of solvent-based acrylic paints in post-war Europe, well before the official date of introduction to the European market of the “Cryla” brand of artist quality paints by George Rowney & Sons in 1963 [3]. In addition, scientific analysis shed light on the variety of traditional and modern pigments and colorants used by Herrera, including: calcite, gypsum, barite, and titanium white (in the form of rutile and anatase); cadmium yellows, oranges, and reds; Pigment Red 83—the synthetic counterpart of natural dye alizarin (1,2-dihydroxyanthraquinone)—precipitated onto an aluminum-rich substrate; viridian and emerald green; cerulean blue, cobalt blue, as well as Prussian blue; iron-containing earths and umber-based pigments; as well as bone and/or ivory black.

**Fig. 1:**
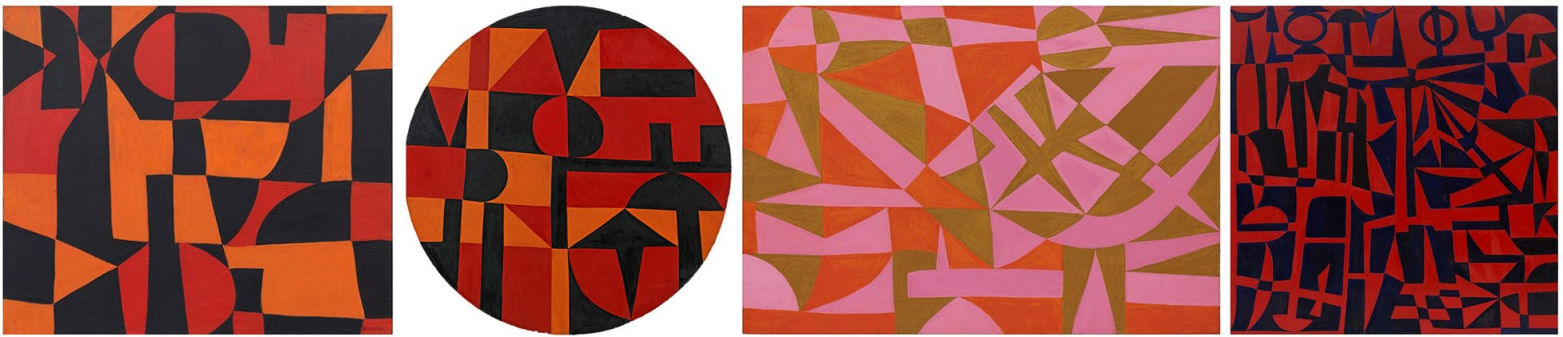
From left to right: *Iberia #25* (1948), catalogued as acrylic on burlap (46 × 54.5 cm; 18 1/8 × 21 ½ inches), private collection, New York; *Iberic* (1949), catalogued as acrylic and oil on canvas glued to pebble board (diameter 101.6 cm; 40 inches), The Metropolitan Museum of Art, New York; *Flights of Colors #16* (1949), catalogued as acrylic on canvas (73 × 106.5 cm; 28 5/8 × 41 7/8 inches), private collection, New York; and *Early Dynasty* (1953), catalogued as acrylic on canvas (121.9 × 121.9 cm; 48 × 48 inches), private collection, New York

Expanding and integrating the authors’ former study of Herrera’s paintings, this article presents a second phase of research aiming to ascertain the possible presence of other early acrylics in the artist’s pre-1963 Parisian work, as well as to explore her materials and painting techniques. A selection of four paintings, namely *Iberia #25* (1948), *Iberic* (1949), *Flights of Colors #16* (1949), and *Early Dynasty* (1953) (Fig. 1), was subjected to an analytical campaign that involved both non-invasive and micro-invasive techniques. Special attention was devoted to the identification of the binding media, pigments, colorants, and extenders employed in Herrera’s creations, as well as to a detailed examination of the painting stratigraphy in an attempt to disclose precious details of her creative process. Moreover, upon donation to The Metropolitan Museum of Art (The Met), *Iberic* underwent in-depth technical imaging along with *Iberia #25*, characterized by analogous geometric and chromatic schemes. Scientific analysis also supported the conservation treatment of *Iberic* in preparation for its display in The Met’s 150th anniversary exhibition *Making The Met, 1870–2020*, whose opening was delayed to August 29th, 2020, due to the Covid-19 pandemic. Besides corroborating a major alteration in the current scholarship on the availability and use of acrylic-based artists’ paints in post-war Europe, this research provides new insights into the choice of materials and studio practice of a groundbreaking Latin American female artist.

## Experimental

The present study entailed the analysis of four paintings (Fig. 1), as follows: *Iberia #25* (1948), catalogued as acrylic on burlap (46 × 54.5 cm; 18 1/8 × 21 ½ inches), private collection, New York; *Iberic* (1949), catalogued as acrylic and oil on canvas glued to pebble board (diameter 101.6 cm; 40 inches), The Metropolitan Museum of Art, New York; *Flights of Colors #16* (1949), catalogued as acrylic on canvas (73 × 106.5 cm; 28 5/8 × 41 7/8 inches), private collection, New York; and *Early Dynasty* (1953), catalogued as acrylic on canvas (121.9 × 121.9 cm; 48 × 48 inches), private collection, New York.

Except for *Iberic*, recently donated to The Met, and *Iberia #25*, shipped to the museum for comparative technical imaging, these works could not be moved from the art gallery or conservation studio where they were on display or under treatment (Lisson Gallery and Cranmer Art Group LLC, New York). As a result, scientific analysis of *Flights of Colors #16* and *Early Dynasty* mostly relied on the removal of samples and the use of micro-invasive techniques available in The Met’s Department of Scientific Research (DSR). Samples were collected from selected areas of the four paintings under study to address various questions regarding the artist’s materials and techniques, as follows (Additional file 1: Fig. S1): 9 from *Iberia #25* (6 scrapings and 3 cross sections); 18 from *Iberic* (9 scrapings and 9 cross sections); 6 from *Flights of Colors #16* (3 scrapings and 3 cross sections); and 9 from *Early Dynasty* (4 scrapings and 5 cross sections). Scrapings were analyzed with Fourier-transform infrared (FTIR) spectroscopy and pyrolysis-gas chromatography/mass spectrometry (Py-GC/MS) to provide a characterization of the binding media. Multi-layered samples were mounted as cross sections, photographed under visible polarized and ultraviolet (UV) illumination, and examined by means of Raman spectroscopy and scanning electron microscopy coupled with energy-dispersive X-ray spectroscopy (SEM/EDS) to study the painting stratigraphy and gain insight into the variety of pigments and extenders used. Surface-enhanced Raman spectroscopy (SERS), performed on microscopic paint scrapings, provided complementary information on the organic colorants and lake pigments present. In addition to micro-invasive analysis, *Iberic* and *Iberia #25* also underwent close visual examination and imaging campaigns by means of normal and raking light, IR and UV illumination, X-radiography, as well as macro-X-ray fluorescence (MA-XRF) spectroscopy. Experimental conditions for the analytical techniques employed are reported below.

### Visual examination

*Iberic* and *Iberia #25* were examined under normal and raking light, IR and UV illumination, and with the aid of a stereomicroscope. IR reflectography (IRR) was carried out by means of an Osiris InGaAs near-IR camera with a 6-element, 150-mm focal length, f/5.6 and f/45 lens. The light source was a pair of Lowel Tota lamps (R7, 120 V, 500 W bulb) on a variable transformer. Images were captured in the 900–1700 nm spectral range. Distance of the camera from the paintings was 95 cm, resulting in a field size of 42 cm with pixel dimensions of 4096 × 4096. Images were processed and stitched together by means of Photoshop. UV photography was conducted by using a Canon 5DS camera with Kodak 2E and Kodak CC40R filters. Illumination was provided by two 4-foot 40 W GE F40BLB lamps (UV-A glass mercury vapor lights with a peak at 365 nm).

### X-radiography

Computed radiography was undertaken for *Iberic* and *Iberia #25* using a TFI Hotshot portable industrial X-ray unit, consisting of 603 head and 805D Control. The system has a 0.5-mm focal spot and 96.5-cm radiation beam. Images were recorded onto Industrex Flex XL Blue 5537 plates and digitized with a Carestream HPX-1 scanner with Industrex software. Plates were exposed at 30 kV and 5 mA for 30 s and scanned at a resolution of 50 μm, equivalent to 508 ppi. Images were processed and stitched together by means of NIP2 mosaicking software.

### MA-XRF

*Iberic* and *Iberia #25* were mapped using a Bruker M6 Jetstream instrument with the X-ray source operated at 50 kV and 0.5 mA. A selected area in *Iberic* and the full *Iberia #25* were scanned with a 580-μm spot size, an 800-μm step size, and a dwell time of 90 ms/pixel. The spectra were processed using the Bruker M6 Jetstream software.

### SEM/EDS

Analysis of cross sections was performed with a FE-SEM Zeiss Σigma HD system equipped with an Oxford Instrument X-MaxN 80 silicon drift detector (SDD). Back-scattered electron (BSE) imaging, as well as EDS elemental analysis and mapping, were carried out in high vacuum at 20 kV and at an 8.5-mm working distance, on 12-nm carbon-coated samples.

### Raman

Analysis was conducted using a Bruker Senterra Raman spectrometer equipped with an Olympus 50× long working distance microscope objective and a charge-coupled device (CCD) detector. A continuous wave diode laser, emitting light at 785 nm, was used as the excitation source, and two holographic gratings (1800 and 1200 rulings/mm) provided a spectral resolution of 3–5 cm^−1^. The output laser power (10 or 25 mW), number of scans, and integration time were adjusted to prevent damage from overheating and according to the response of the various areas and samples examined. Spectra were interpreted by comparison with published data and library databases available at The Met’s DSR.

### SERS

Analysis was carried out by means of the same Raman instrument described above, in association with an Olympus 20× long working distance microscope objective and a Spectra Physics Cyan solid state laser providing excitation at 488 nm. A two-step procedure was applied to the samples prior to analysis in order to account for the possible presence of both free dyes and lake pigments [4]. In detail, each sample was initially analyzed as is, without any pretreatment, after being covered with a 2 μL droplet of silver colloid and 0.5 μL of 0.5 M potassium nitrate—the first amplifying the inherently weak Raman signal and the latter serving as aggregating agent for the nanoparticles. Upon rinsing with ultrapure water, samples were then exposed to hydrofluoric acid (HF) vapor in a polyethylene micro-chamber for 5 min and analyzed again. Silver colloids were prepared by microwave-supported glucose reduction of silver sulfate in the presence of sodium citrate as a capping agent, following a synthesis previously published [5]. An output laser power of 4 mW was employed for the analysis, with two integrations of 30 s. Spectra were interpreted by comparison with published data and library databases available at The Met’s DSR.

### FTIR

Analysis was performed with a Hyperion 3000 FTIR spectrometer equipped with a mercury cadmium telluride (MCT) detector. Each sample was crushed in a Spectra Tech diamond anvil cell and analyzed through a 15× objective in transmission mode. Spectra were collected at a resolution of 4 cm^−1^ and obtained as the sum of 128 or 256 scans, depending on the response of the various areas or samples examined. Spectra were interpreted by comparison with published data and library databases available at The Met’s DSR.

### Py-GC/MS

Analysis was conducted on an Agilent 6890 gas chromatograph equipped with a Frontier PY-2020iD Double-Shot vertical furnace pyrolyzer fitted with an AS-1020E Auto-Shot autosampler. The GC was coupled to a 5973N single quadrupole mass selective detector (MSD). Samples of 30–50 µg were weighed out in deactivated pyrolysis sample cups (PY1-EC80F Disposable Eco-Cup LF) on a Mettler Toledo UMX2 Ultra microbalance. Samples were then either pyrolyzed without derivatization or derivatized with tetramethylammonium hydroxide (TMAH) before pyrolysis. Derivatization took place in the same cups as follows: 3–4 µL of 25% TMAH in methanol (both from Fisher Scientific), depending on the sample size, were added directly to the sample in each cup with a 50-µL syringe and, after 1 min, loaded onto the autosampler. The interface to the GC was held at 320 °C and purged with helium for 30 s before opening the valve to the GC column. The samples were then dropped into the furnace and pyrolyzed at 550°C for 30 s. The pyrolysis products were transferred directly to a DB-5MS capillary column (30 m × 0.25 mm × 1 µm) with the helium carrier gas set to a constant flow of 1.5 mL/min. Injection with a 30:1 split was used, in accordance with the sample size. The GC oven temperature program was: 40°C for 1 min; 10°C/min to 320°C; isothermal for 1 min. The Agilent 5973N MSD conditions were set as follows: transfer line at 320°C, MS Quad 150°C, MS Source 230°C, electron multiplier at approximately 1770 V; scan range 33–550 amu. For samples run with TMAH, the detector was turned off until 3 min to avoid saturation by excess of derivatizing agent and solvent. Data analysis was performed on an Agilent MSD ChemStation D.02.00.275 software by comparison with the NIST 2005 spectral libraries.

### Preparation and examination of cross sections

Cross sections were prepared by embedding each sample within a double layer of methyl methacrylate resin (Technovit® 2000 LC). Each layer of resin was cured under UV light for 20 min. Excess resin was ground off and the surface was finely polished using CarbiMet 2 and Micro-mesh abrasive paper of various grits to expose the sample’s stratigraphy. Cross sections were examined by means of a Zeiss Axio Imager M2m microscope, equipped with an Axiocam HRc digital camera and providing 50×, 100×, 200×, 400×, and 500× magnification. Photographs were collected using the AxioVision 4.X.X software.

## Results and discussion

A summary of the results obtained from the scientific analysis of the four paintings under study is reported in Table 1. These data are presented and discussed in further detail in the following paragraphs.

**Table 1:** A summary of the results obtained from the scientific analysis of the four paintings under study

Title and date	Samples	Micro-invasive techniques	Ground layer	Paint layers	Binding media or coatings*
*Iberia #25* (1948)	S1) Scraping of black paint	Py-GC/MS			*n*-BMA, linseed oil, Pinaceae resin
	S2) Scraping of black paint	Py-GC/MS			Oil (possibly linseed) with increased azelaic acid, Pinaceae resin, trace *n*-BMA
	S3) Cross section of black paint	Optical microscopy, SEM/EDS, Raman	Titanium white (rutile), anhydrite, talc	Cerulean blue, Prussian blue, bone or ivory black, cadmium sulfoselenide	
	S4) Scraping of red paint	Py-GC/MS			Oil with increased palmitic acid or decreased azelaic acid, unsaturated fatty acids, phthalic acid di(3-ethylphenyl)ester, Pinaceae resin
	S5) Scraping of pink wash	Py-GC/MS			Oil (possibly linseed) with increased azelaic acid, unsaturated fatty acids, Pinaceae resin
	S6) Cross section of red paint	Optical microscopy, SEM/EDS, Raman, SERS	Titanium white (rutile), anhydrite, talc	Prussian blue, cerulean blue, bone or ivory black, cadmium sulfoselenide, purpurin lake, calcium sulfate, barite, zinc white	
	S7) Scraping of red paint	Py-GC/MS			Oil (possibly linseed) with increased azelaic acid, Pinaceae resin
	S8) Cross section of orange paint	Optical microscopy, SEM/EDS, Raman, SERS	Titanium white (rutile), anhydrite, talc	Prussian blue, bone or ivory black, cadmium sulfoselenide, cadmium yellow, purpurin lake, alumina, calcium carbonate	
	S9) Scraping of orange paint	Py-GC/MS			*n*-BMA, oil (possibly linseed) with decreased azelaic acid, unsaturated fatty acids, Pinaceae resin
*Iberic* (1949)	S1) Scraping of black paint	Py-GC/MS, FTIR			*n*-BMA, oil, Pinaceae resin, trace phthalic anhydride, phthalates, and styrene
	S2) Scraping of orange paint	Py-GC/MS, FTIR			*n*-BMA, oil, Pinaceae resin, trace phthalic anhydride, phthalates, and styrene
	S3) Scraping of red paint	Py-GC/MS, FTIR			*n*-BMA, oil, Pinaceae resin, trace phthalic anhydride, phthalates, and styrene
	S4) Cross section of black paint	Optical microscopy, SEM/EDS, Raman	Titanium white (rutile), anhydrite, talc	Bone or ivory black, cadmium yellow, Cd- and Se-containing red particles	
	S5) Cross section of red paint	Optical microscopy, SEM/EDS, Raman	Titanium white (rutile), anhydrite, talc	Bone or ivory black, cadmium sulfoselenide, barite	
	S6) Cross section of orange paint	Optical microscopy, SEM/EDS, Raman	Titanium white (rutile), anhydrite, talc	Bone or ivory black particles, cadmium sulfoselenide	
	S7) Cross section of red paint	Optical microscopy, SEM/EDS, Raman	Titanium white (rutile), anhydrite, talc	Cadmium sulfoselenide	
	S8) Cross section of gray wash	Optical microscopy, SEM/EDS, Raman	Titanium white (rutile), anhydrite, talc		
	S9) Cross section of orange paint	Optical microscopy, SEM/EDS, Raman	Titanium white (rutile), anhydrite, talc	Cadmium sulfoselenide, traces of black layer	
	S10) Cross section of orange paint	Optical microscopy, SEM/EDS, Raman	Titanium white (rutile), anhydrite, talc	Cadmium sulfoselenide	
	S11) Cross section of gray paint	Optical microscopy, SEM/EDS, Raman	Titanium white (rutile), anhydrite, talc		
	S12) Scraping of coating on top of black paint	Py-GC/MS, FTIR			Oil with increased azelaic acid, *n*-BMA, phthalic acid, trace Pinaceae resin and styrene
	S13) Scraping of gummy accretions on surface	Py-GC/MS, FTIR			2-ethylhexyl acrylate polymer
	S14) Scraping of coating on top of red paint	Py-GC/MS			*n*-BMA, various other acrylics, drying oil, trace Pinaceae resin
	S15) Scraping of coating on top of orange paint	Py-GC/MS			*n*-BMA, various other acrylics, drying oil, trace Pinaceae resin
	S16) Scraping of coating on top of orange paint	Py-GC/MS			*n*-BMA, various other acrylics, drying oil, trace Pinaceae resin
	S17) Cross section of red paint	Optical microscopy			
	S18) Scraping of coating on top of red paint	Py-GC/MS			*n*-BMA, various other acrylics, drying oil, trace Pinaceae resin
*Flights of Colors #16* (1949)	S1) Scraping of pink paint	Py-GC/MS			Oil (possibly walnut, linseed mixed with poppy, or linseed with increased palmitic acid), unsaturated fatty acids, Pinaceae resin
	S2) Cross section of pink paint	Optical microscopy, SEM/EDS, Raman, SERS	Titanium white (rutile), anhydrite, talc	Yellow ocher, cadmium yellow, viridian, purpurin lake, zinc white	
	S3) Scraping of orange paint	Py-GC/MS			Oil (possibly walnut, linseed mixed with poppy, or linseed with increased palmitic acid), Pinaceae resin, trace *n*-BMA
	S4) Cross section of orange paint	Optical microscopy, SEM/EDS, Raman, SERS	Titanium white (rutile), anhydrite, talc	Iron(III) oxide, purpurin lake, cadmium red, cadmium orange, cadmium yellow, barite	
	S5) Scraping of brown paint	Py-GC/MS			Oil (possibly walnut or linseed mixed with poppy), unsaturated fatty acids
	S6) Cross section of brown paint	Optical microscopy, SEM/EDS, Raman	Titanium white (rutile), anhydrite, talc	Cadmium red, iron(III) oxide, yellow ocher, viridian, bone or ivory black, zinc white, cobalt blue, Prussian blue	
*Early Dynasty* (1953)	S1) Scraping of blue paint	Py-GC/MS			Oil (possibly linseed) with increased azelaic acid, Pinaceae resin
	S2) Cross section of blue paint	Optical microscopy, SEM/EDS, Raman	Titanium white (rutile), anhydrite, talc	Cobalt blue, Prussian blue, cerulean blue	
	S3) Scraping of blue paint	Py-GC/MS			Oil (possibly linseed) with increased azelaic acid, Pinaceae resin
	S4) Cross section of blue paint	Optical microscopy, SEM/EDS, Raman	Titanium white (rutile), anhydrite, talc	Cobalt blue, Prussian blue, cerulean blue	
	S5) Cross section of blue paint	Optical microscopy, SEM/EDS, Raman	Titanium white (rutile), anhydrite, talc	Cobalt blue, Prussian blue, cerulean blue	
	S6) Cross section of blue paint	Optical microscopy, SEM/EDS, Raman	Titanium white (rutile), anhydrite, talc	Cobalt blue, Prussian blue, cerulean blue, ultramarine blue	
	S7) Scraping of blue paint	Py-GC/MS			
	S8) Scraping of red paint	Py-GC/MS			Oil with increased palmitic acid
	S9) Cross section of red paint	Optical microscopy, SEM/EDS, Raman	Titanium white (rutile), anhydrite, talc	Cadmium sulfoselenide	

*This column indicates the binding media found in the paint layers, as well as, in the case of samples S12, S14, S15, S16, and S18 from *Iberic*, the organic coatings that appear to have been applied on top of the paint

### *Iberia #25* (1948)

**Fig. 2:**
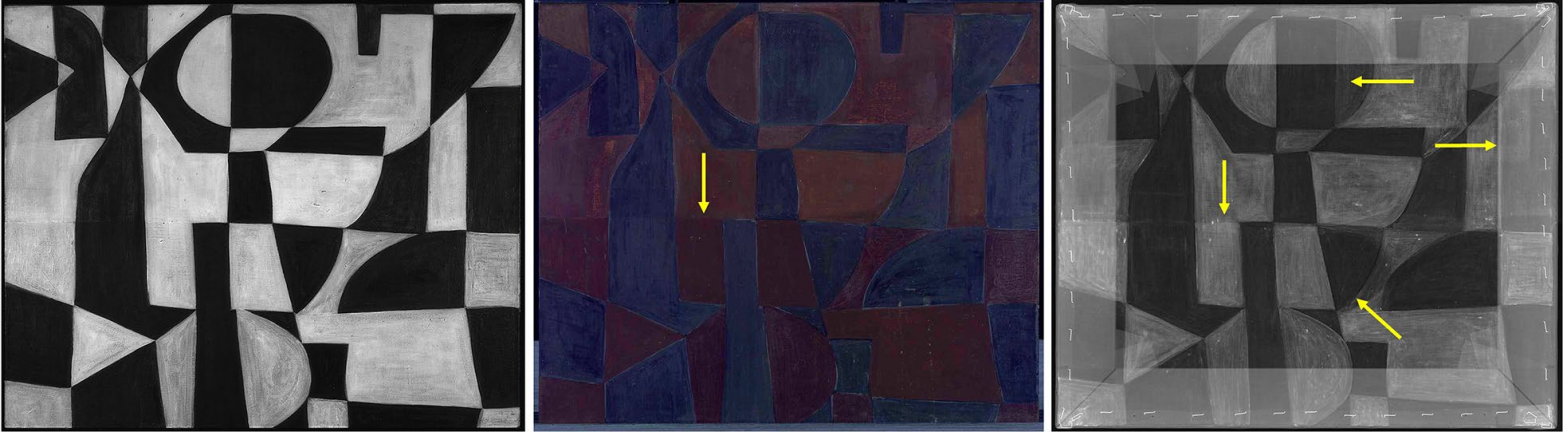
From left to right: IR reflectogram, UV photograph, and X-radiograph of *Iberia #25* (1948). Arrows indicate compositional changes and varying paint opacities discussed in the text

Examination of *Iberia #25* by means of IRR did not reveal any discernible underdrawing (Fig. 2). Given that each red and orange field of color in this work is adjacent to one or multiple black fields, any existing pencil lines were likely obscured by the artist while following, and covering, her drawn outlines with black paint, thus making them invisible to IRR. Nevertheless, certain areas of lighter opacity, visible in the reflectogram in some of the red and orange fields in the bottom half of the painting, suggest either the overlaying of red and orange colors in those areas, or the use of red and orange paints of different composition within the same painting. This opacity shift is even more prominently evident under UV illumination (Fig. 2). In the corresponding image, the work appears to be bisected along its center horizontal axis, and all fields in the bottom portion, whether currently orange or red, appear as a darker red. A large red color field in the upper right part of the composition also has a similar darker hue under UV illumination. Another distinctive feature observed under UV light is a milky greenish fluorescence that displays a distinctive brush application pattern within all black fields, including uneven build-up edges visible along the perimeter of each black field typical of the extra material pooling up at the outer edge of the brush. This pattern may indicate the presence of additional medium mixed into the paint or the local brush application of a coating.

A similar horizontal bisection of the *Iberia #25* composition is visible in the X-radiograph (Fig. 2), where the orange and red fields in the bottom half of the work are generally more radio-opaque than those in the upper half, also suggesting the presence of different pigments either within or under the main paint layer. The X-radiograph also reveals that Herrera made a few changes to the composition. Most significant is the modification of the large “D-like” black shape at top center, which the artist had first painted as a large rectangle surrounded by a semicircle of different color. The other two alterations are more subtle: the trimming of a curving profile at bottom center and the elimination of a small rectangular shape along the right side in the upper half of the painting.

**Fig. 3:**
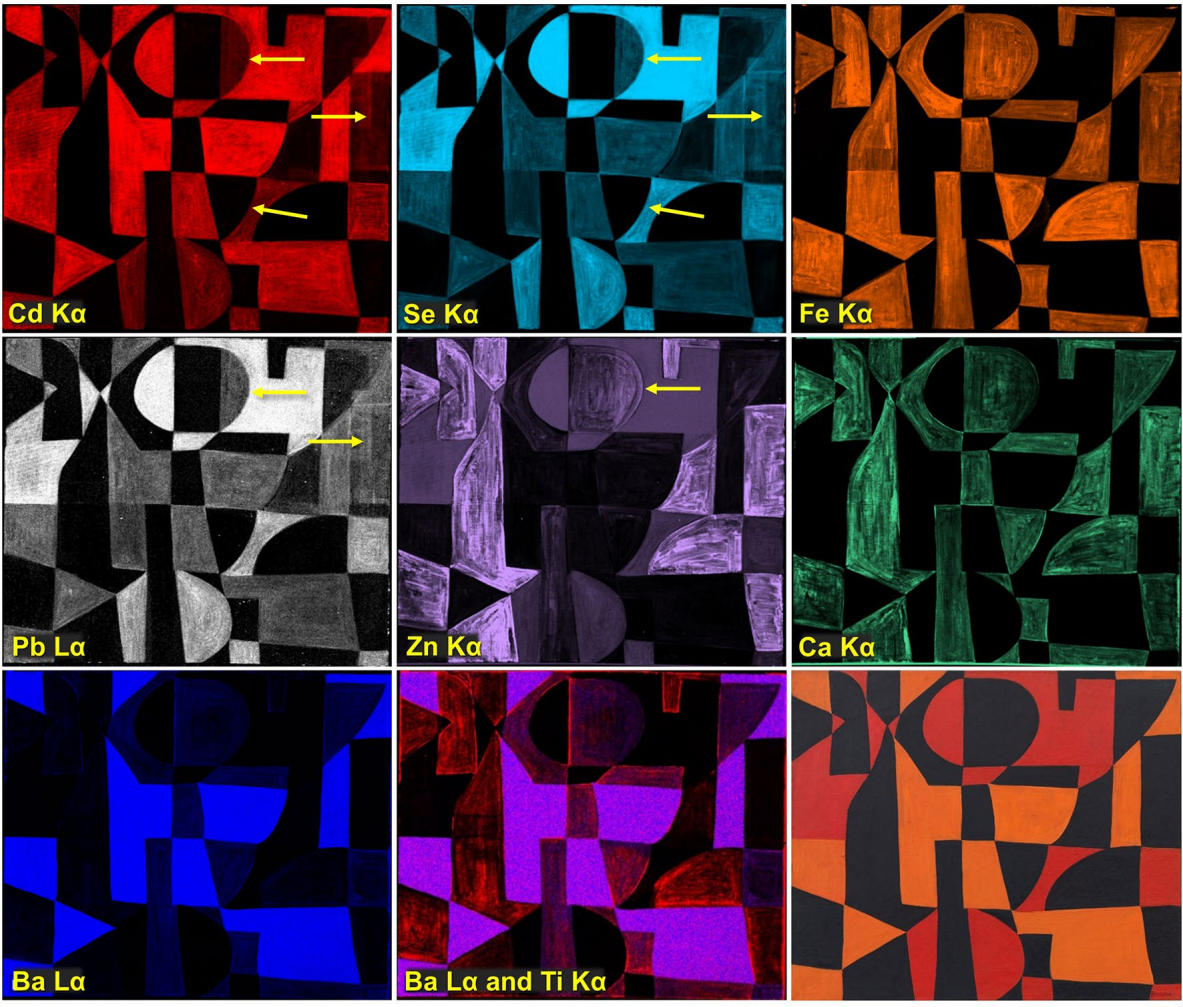
Elemental distribution maps of *Iberia #25* (1948) obtained by MA-XRF: Cd Kα, Se Kα, Fe Kα, Pb Lα, Zn Kα, Ca Kα, Ba Lα, and composite Ba Lα (blue) and Ti Kα (red). The painting is also shown at bottom right for comparison. Arrows indicate compositional changes discussed in the text

The elemental distribution maps obtained by MA-XRF show the locations of the main components in the red, orange, and black paints and reveal details of the brushwork (Fig. 3). In areas of red paint, cadmium (Cd) and selenium (Se) co-locate, confirming that cadmium red (CdS.xCdSe) is present. In orange areas, Cd is the main component, indicating that cadmium yellow (CdS) was used; relatively weaker signals for Se are also detected, pointing to the possible presence of a cadmium red-containing paint in a layer below the orange and/or to the admixing of some cadmium red with cadmium yellow in the orange paint (Fig. 3 and Additional file 2: Fig. S2). The distribution of lead (Pb) aligns with that of Se, indicating that a Pb-containing compound is located in the red paint. The map of barium (Ba) shows that this element is mostly present in the orange paint (Additional file 3: Fig. S3). In the black paint areas, calcium (Ca) and iron (Fe) are detected; however, the distribution of these two elements is not identical. SEM/EDS and Raman analysis of cross section S3, taken from a black area and discussed in detail below, indicates that a bone or ivory black, characterized by the presence of Ca and phosphorus (P), is mixed with Prussian blue in the black paint at the top of the stratigraphy and that paint that only contains Prussian blue was applied below the black paint layer. As also found upon analysis of the cross sections, Ca is present in the ground layer of all samples investigated. No Fe-containing compounds other than Prussian blue were identified in any of the specimens removed from this painting. The zinc (Zn) and, to a lesser extent, the Fe distribution maps reflect the complexity of the layering and mixing of paints. Therefore, a detailed microscopic examination and analysis of paint cross sections was necessary to gain further insight into the paint stratigraphy.

The Cd Kα, Se Kα, Pb Lα, and Zn Kα MA-XRF distribution maps (Fig. 3) also display some of the compositional changes visible in the X-radiograph. The distributions of Cd and Se suggest that the small semicircle shape described above may have been originally painted red before being changed to black and merged with the black rectangle to its left to form the large “D-like” field in the upper center. A similar change can be visualized for the smaller rectangle present along the right edge of the composition. Around the mid-center, the profile of what appears as a less radio-opaque “D” in the X-radiograph (Fig. 2) was trimmed to a rounded triangular field by painting with cadmium red paint; however, this underlying, less radio-opaque paint did not register in the elemental distribution maps.

**Fig. 4:**
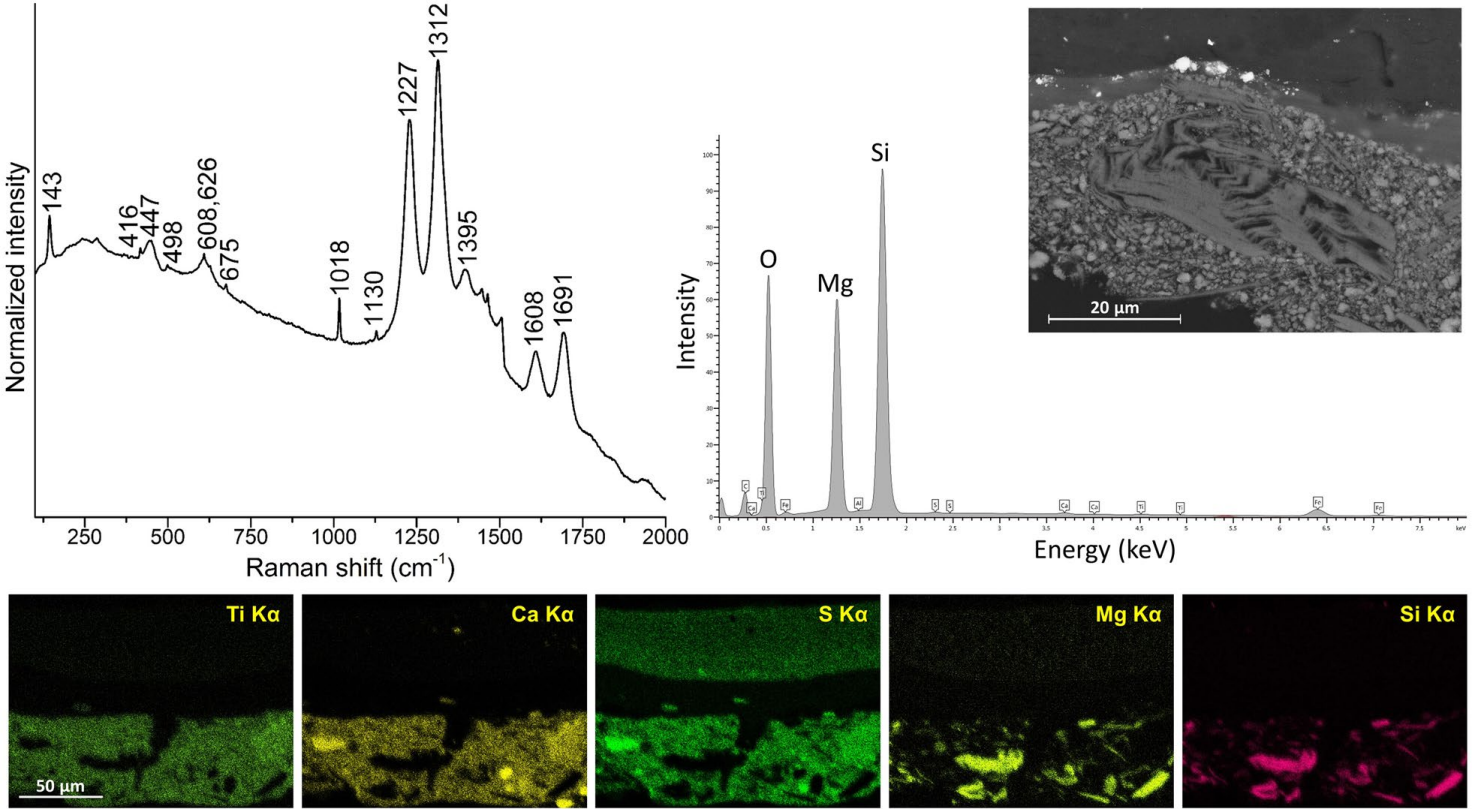
Data collected from the ground layer of cross section S6 from *Iberia #25* (1948)*.* Top left, Raman spectrum, displaying the distinctive bands of rutile at 143, 447, 608 cm^−1^ and of anhydrite at 416, 498, 608, 626, 675, 1018, 1130 cm^−1^. Broad features at ca. 1227, 1312, 1395, 1608, 1691 cm^−1^ are attributed to the luminescence emission of Nd^3+^ of composite titanium dioxide pigments produced by co-precipitation with BaSO_4_ or CaSO_4_. Top right, EDS spectrum collected from an area in the left portion of S6, along with the corresponding BSE image showing the typical sheet structure of talc. Bottom, EDS elemental maps of Ti Kα, Ca Kα, S Kα, Mg Kα, and Si Kα, acquired from the central portion of S6

Inspection under a microscope of samples S3, S6, and S8, i.e. cross sections removed in areas of black, red, and orange paint, respectively, showed the presence of a preparatory layer with a thickness ranging between 10 and 60 μm. SEM/EDS and Raman analyses showed that this ground layer consists of a mixture of titanium white, calcium sulfate, and talc (Fig. 4). Titanium white is present in the tetragonal rutile form, with characteristic Raman bands at ca. 143, 447, and 608 cm^−1^, while the degree of hydration of the calcium sulfate component is ascertained from the detection of the distinctive signals at ca. 416, 498, 608, 626, 675, 1018, and 1130 cm^−1^, assigned to anhydrite. In all Raman spectra acquired from the preparatory layer, a combination of broad features at ca. 1227, 1312, 1395, 1608, and 1691 cm^−1^, attributed to the luminescence emission of Nd^3+^, is indicative of composite titanium dioxide pigments produced since the early 1940s by co-precipitation with BaSO_4_ or CaSO_4_ [6]. The same spectral pattern was recently observed in samples from other modern paintings and sculptures, including a work dated to 1932 by Alexander Calder [7]. Calder also lived in France until 1933 and this unusual similarity may suggest that both artists could have acquired this material from a common source.

**Fig. 5:**
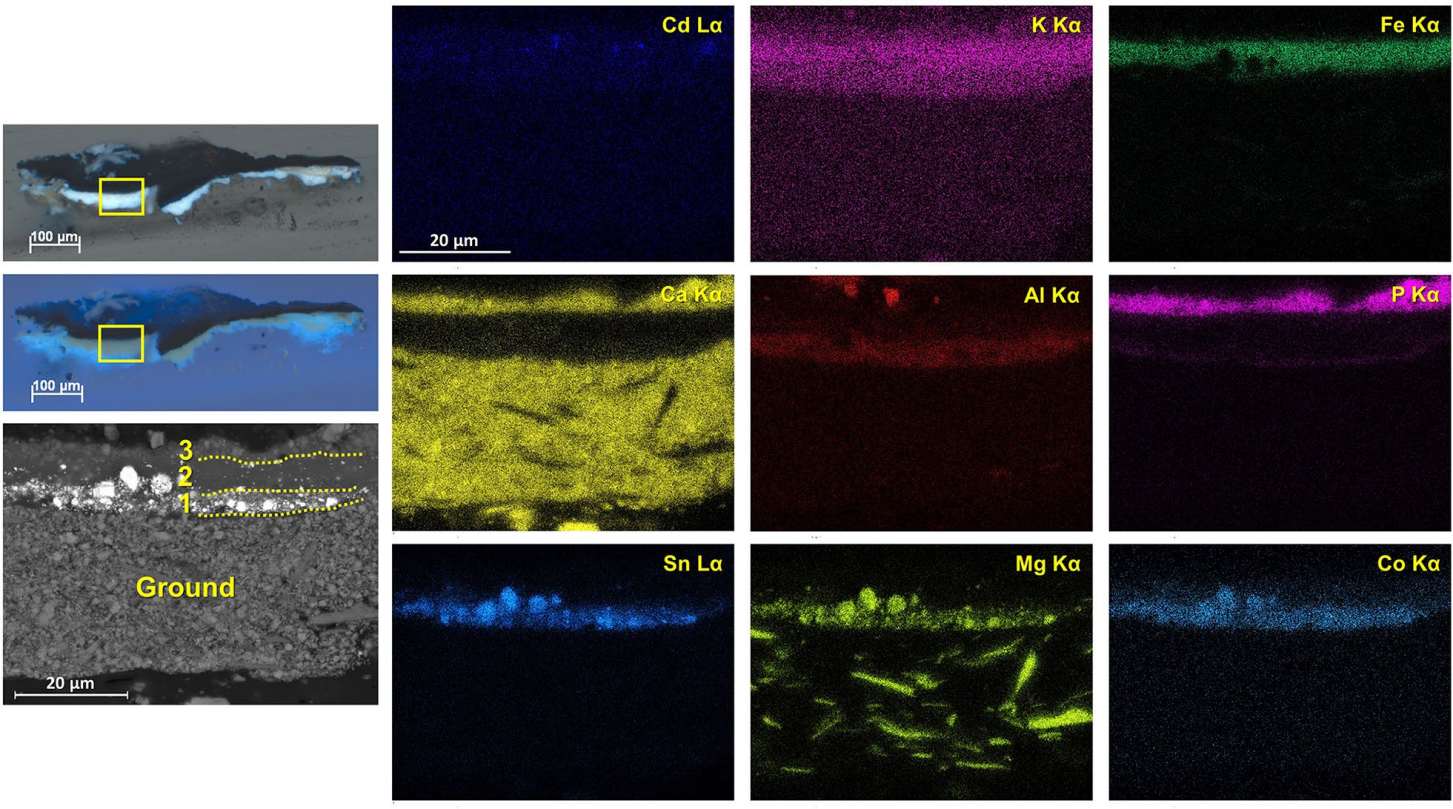
Left, polarized light and UV light microphotographs of cross section S3 from *Iberia #25* (1948), with BSE image of a portion of the sample indicated by a yellow rectangle. Right, EDS elemental maps of Cd Lα, K Kα, Fe Kα, Ca Kα, Al Kα, P Kα, Sn Lα, Mg Kα, and Co Kα

**Fig. 6:**
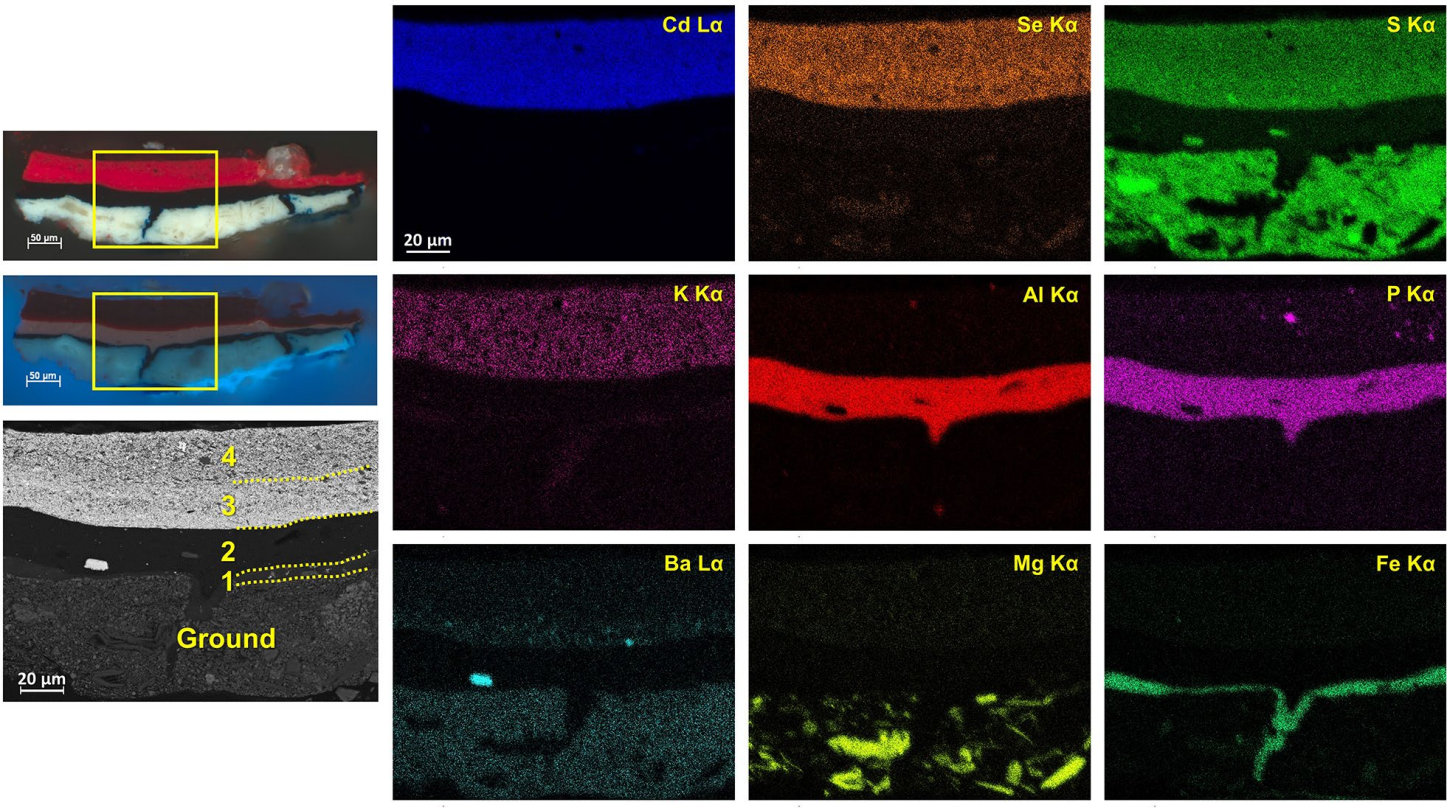
Left, polarized light and UV light microphotographs of cross section S6 from *Iberia #25* (1948), with BSE image of a portion of the sample indicated by a yellow rectangle. Right, EDS elemental maps of Cd Lα, Se Kα, S Kα, K Kα, Al Kα, P Kα, Ba Lα, Mg Kα, and Fe Kα

**Fig. 7:**
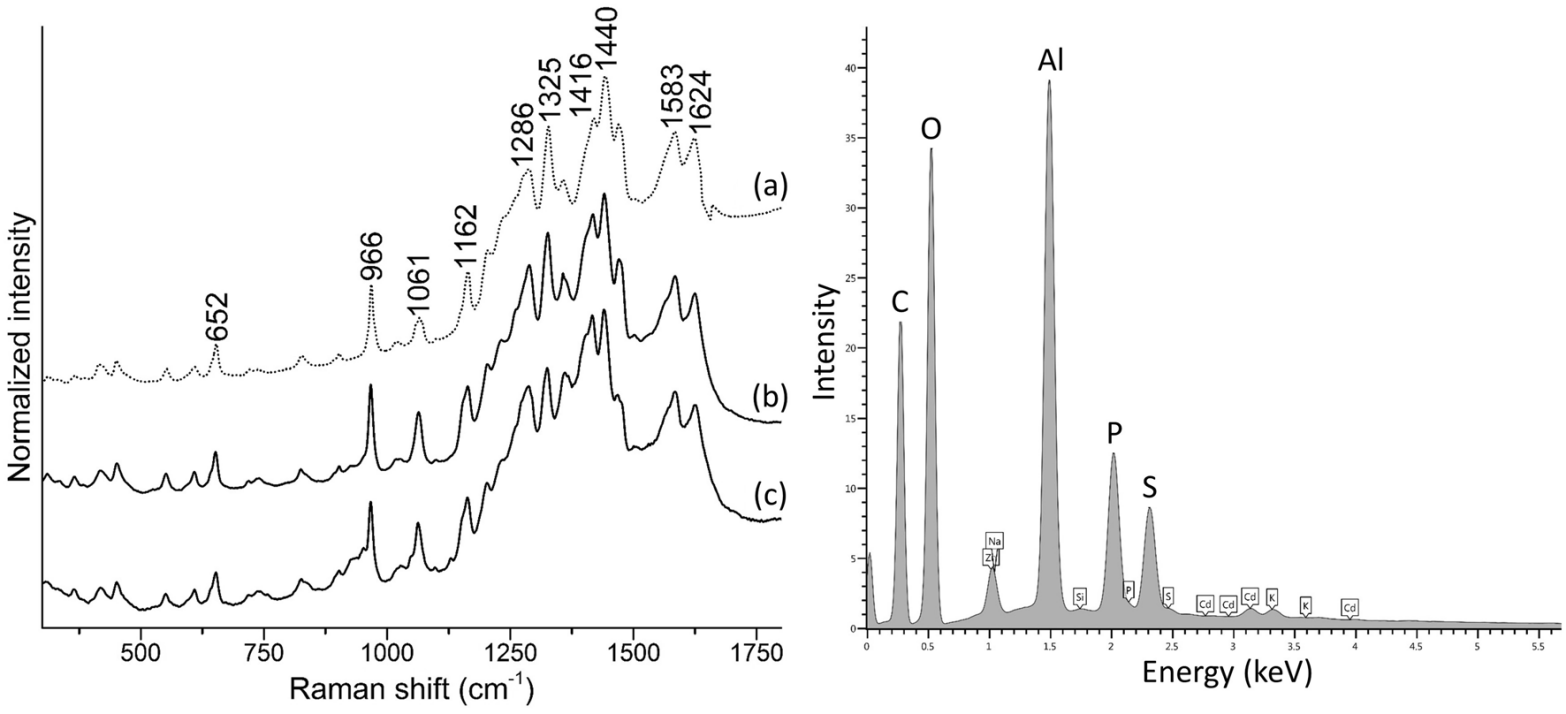
Left, SERS spectra of **a** a purpurin lake synthesized in the laboratory, **b** sample S6 from *Iberia #25* (1948)*,* and **c** sample S2 from *Flights of Colors #16* (1949), all acquired upon HF hydrolysis, displaying characteristic bands at ca. 652, 966, 1061, 1162, 1286, 1325, 1416, 1440, 1583, and 1624 cm^−1^. Right, EDS spectrum of the purpurin lake in sample S6 from *Iberia #25* (1948), exhibiting intense Al, P, and S peaks

**Fig. 8:**
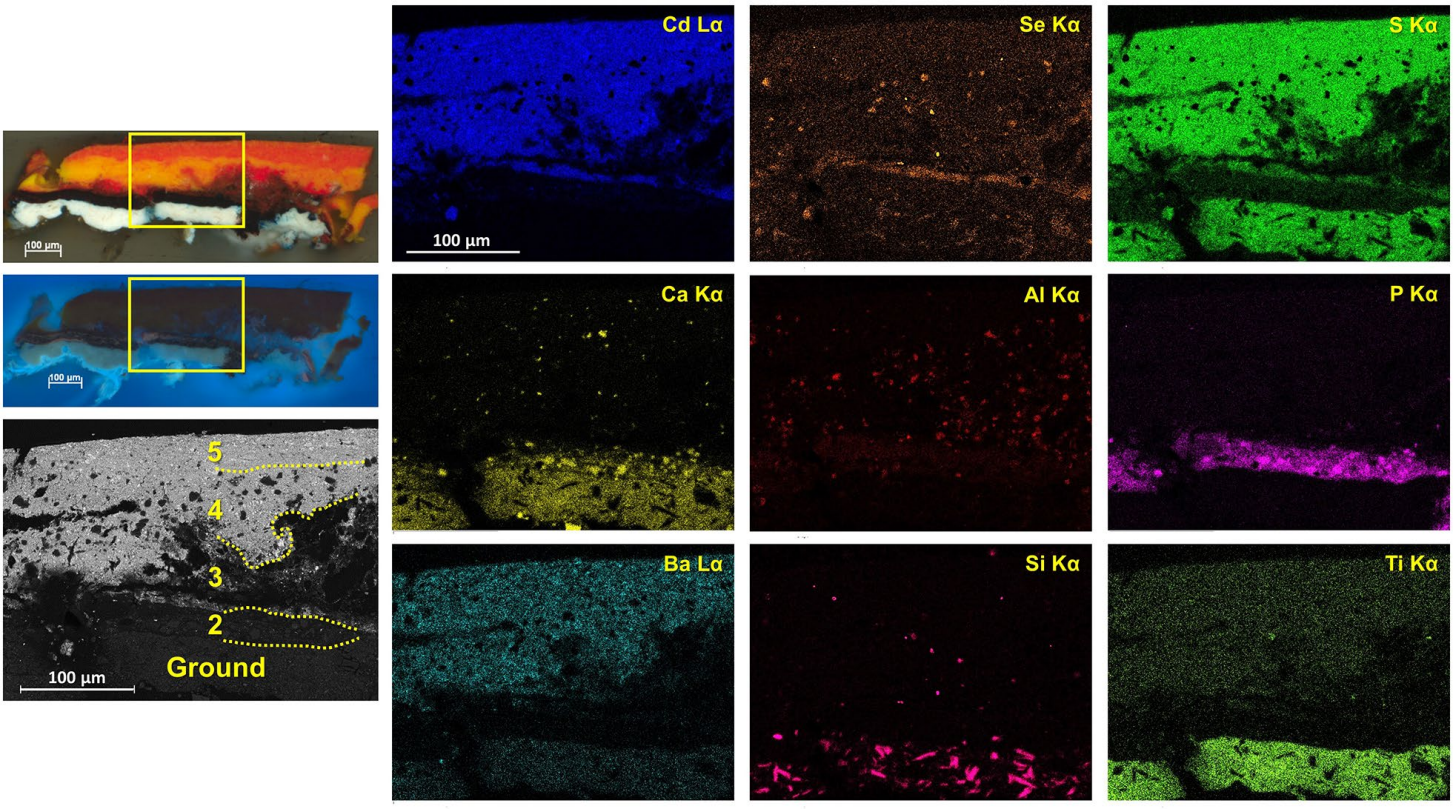
Left, polarized light and UV light microphotographs of cross section S8 from *Iberia #25* (1948), with BSE image of a portion of the sample indicated by a yellow rectangle. Right, EDS elemental maps of Cd Lα, Se Kα, S Kα, Ca Kα, Al Kα, P Kα, Ba Lα, Si Kα, and Ti Kα

Visible polarized light and UV light examination of the cross sections revealed multiple paint layers below each monochrome area. The presence of overpaint is a common feature of most specimens in this study and may indicate subsequent alterations of the initial color scheme, although Herrera’s intention to obtain specific hues and visual effects by overlapping different colors is also a possibility. The relationship of colors is especially important to Herrera where, in this case, reds and oranges are different from one another. She reportedly manipulated the hues on her palette by mixing and/or superimposing different colors to achieve a satisfactory color balance within her compositions.^3^ In cross section S3, for instance, three distinct paint layers of comparable thickness were applied over the ground, as shown by microphotographs and EDS elemental maps (Fig. 5): from bottom to top, the first mostly consists of magnesium (Mg)-rich cerulean blue; the second contains Prussian blue, along with a few particles of cerulean blue and cadmium sulfoselenide, likely a residue from an uncleaned brush or palette; and the third is based on a bone or ivory black mixed with Prussian blue. Cross section S6 comprises four paint layers (Fig. 6), as follows: a thin layer of Prussian blue with very few particles of cerulean blue and cadmium sulfoselenide; a mixture of aluminum (Al)-, P-, and sulfur (S)-rich pigment with a bone or ivory black, calcium sulfate, and barite, as well as possibly relatively low amounts of zinc white; and two applications of a cadmium sulfoselenide pigment with a red hue, the topmost of which appears to be mixed with small amounts of a bone or ivory black. While it was not possible to identify the Al-, P-, and S-rich pigment using Raman spectroscopy due to its strong fluorescence emission, the EDS elemental composition and pink UV-induced autofluorescence suggest that it is likely an organic lake. Accordingly, SERS spectra collected upon HF treatment are dominated by intense bands at ca. 652, 966, 1061, 1162, 1286, 1325, 1416, 1440, 1583, and 1624 cm^−1^ (Fig. 7). This spectral pattern is consistent with that observed for a reference purpurin lake sample synthesized in the laboratory by precipitating a solution containing purpurin and sodium hydroxide on aluminum sulfate [8]. Cross section S8 also displays a complex stratigraphy, in which boundaries between adjacent layers are not always well defined (Fig. 8). This suggests that Herrera may have reworked the area while the paint was still wet, dragging it along with the brush. In this case, a first, discontinuous, very thin layer of Prussian blue was applied directly on top of the ground preparation and then overpainted multiple times with the following materials: a dark organic lake displaying a pink UV-induced autofluorescence—possibly the same compound detected in sample S6—albeit combined with relative larger amounts of a bone or ivory black and a few cadmium sulfoselenide particles; a cadmium sulfoselenide pigment with a red hue; cadmium yellow with an alumina filler and calcium carbonate, likely in the form of calcite; and a mixture of Cd-containing pigments.

Unless otherwise specified, the identification of most materials was inferred from the co-localization of elements in the SEM/EDS maps. The presence of cadmium yellow in all orange and red layers was confirmed by the detection of its typical Raman bands at ca. 300 and 600 cm^−1^ (Additional file 4: Fig. S4). In Cd-containing paints, Ba is mostly detected by EDS in individual particles and separately from Cd, suggesting that a Ba-containing material such as barite may be mixed in as a filler. Synthetic organic pigments, sometimes reported in combination with oil and early acrylic Cd-based colors [9], were not detected by Raman analysis in any of the Cd-containing layers in *Iberia #25* or other paintings in this study. Additionally, the use of Prussian blue was verified by the presence of its characteristic Raman bands at ca. 280, 535, 2091, 2121, and 2152 cm^−1^ (Additional file 4: Fig. S4). Also, a bone or ivory black was suggested through a combined identification of broad Raman bands around 1335 and 1595 cm^−1^ and Ca and P in SEM/EDS data (Additional file 4: Fig. S4). The Pb- and Zn-containing compounds observed by MA-XRF could not be identified by Raman spectroscopy and SEM/EDS in the paint samples studied, likely due to their presence in amounts below the detection limits of these two techniques.

**Fig. 9:**
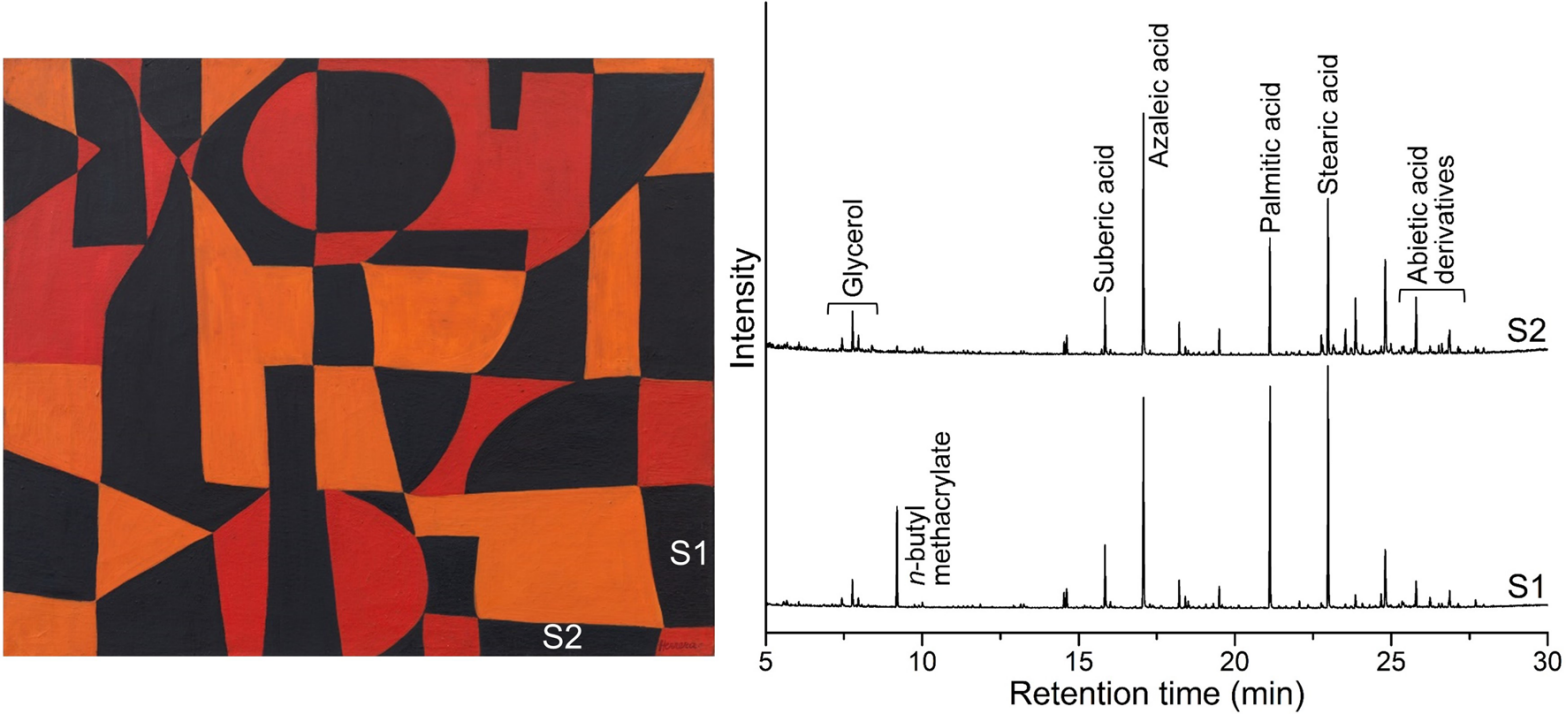
Left, Herrera’s *Iberia #25* (1948), with indication of the sampling location for binding media analysis of the black paint. Right, Py-GC/MS chromatograms obtained upon derivatization with TMAH, showing that while S2 is based on oil and Pinaceae resin, S1 also contains *n*-BMA

Py-GC/MS analysis highlighted the use of different binders in the specimens examined. Sample scrapings S1 and S2, removed from two distinct black fields in the proper left bottom quadrant, appear to contain acrylic and oil binders. The black paint in S1 fluoresces under UV illumination and is mostly composed of *n*-butyl methacrylate (*n*-BMA) in combination with oil and Pinaceae resin (Fig. 9). While multiple paint layers are a possibility as the stratigraphy in this location is unknown, this might also be an example of the miscibility of early solvent-based acrylics with oil paint. In 1947, Magna®, a brand name for a series of acrylic paints prepared from pigments dispersed in *n*-BMA resins and diluted with turpentine, mineral spirits, xylenes, and toluene, became available in the United States, but it is not known to have been released in Europe. The first manufacturer to introduce artists' acrylic paints in Europe—a water-based emulsion sold under the brand name “Cryla”—was George Rowney in 1963 [3]. In practical terms, Magna® dried quickly by evaporation of the organic solvent; it remained resoluble in many hydrocarbon solvents, as well as further layers of paint, and could be blended with oil paint [10–12]. In contrast, the drying process of emulsion paints involves a complicated coalescence of emulsified polymer spheres after an initial evaporation of water. These paints become insoluble in water after they have dried and can be painted over with oil or acrylic emulsion paint [13]. In S1, the palmitic to stearic acid (P/S = 0.9) and azelaic to palmitic acid (A/P = 1.0) ratios are consistent with linseed oil. The binder in S2, on the other hand, was found to be an oil, possibly also linseed, but with elevated azelaic acid levels (P/S = 0.7, A/P = 2.2), indicating accelerated drying of the oil paint. Pinaceae resin is also present in this specimen, along with a trace of *n*-BMA, possibly due to carry-over on the paint brush. Sample scrapings S4, S5, and S7, removed from various red areas, are oil-based paints. In detail, S4 appears to contain an oil, possibly with added heavy metal palmitates or decreased azelaic acid (P/S = 2.5, A/P = 0.3), and in which the detection of unsaturated fatty acids suggests incomplete drying. A small amount of phthalic acid, di(3-ethylphenyl)ester, was also identified, possibly serving as a plasticizer, as is Pinaceae resin. The red paint in S7, fluorescent under UV illumination, is a different kind of oil paint, possibly linseed, with an elevated azelaic acid content (P/S = 0.8, A/P = 2.3), reflecting accelerated drying, and also containing Pinaceae resin. The fluorescing pink paint in S5 is similar: an oil paint, possibly linseed, with increased azelaic acid amount (P/S = 1.0, A/P = 1.9). Unsaturated fatty acids and Pinaceae resin are also present. Conversely, sample S9, removed from an area of orange paint, has a binder composed of *n-*BMA with oil, possibly linseed, with hindered drying that decreased the azelaic acid amount (P/S = 1.0, A/P = 0.5). Unsaturated fatty acids and Pinaceae resin were also detected. As the stratigraphy of this sample is also unknown, this could possibly be another example of the miscibility of early solvent-based acrylic paints with oil paints.

### *Iberic *(1949)

**Fig. 10:**
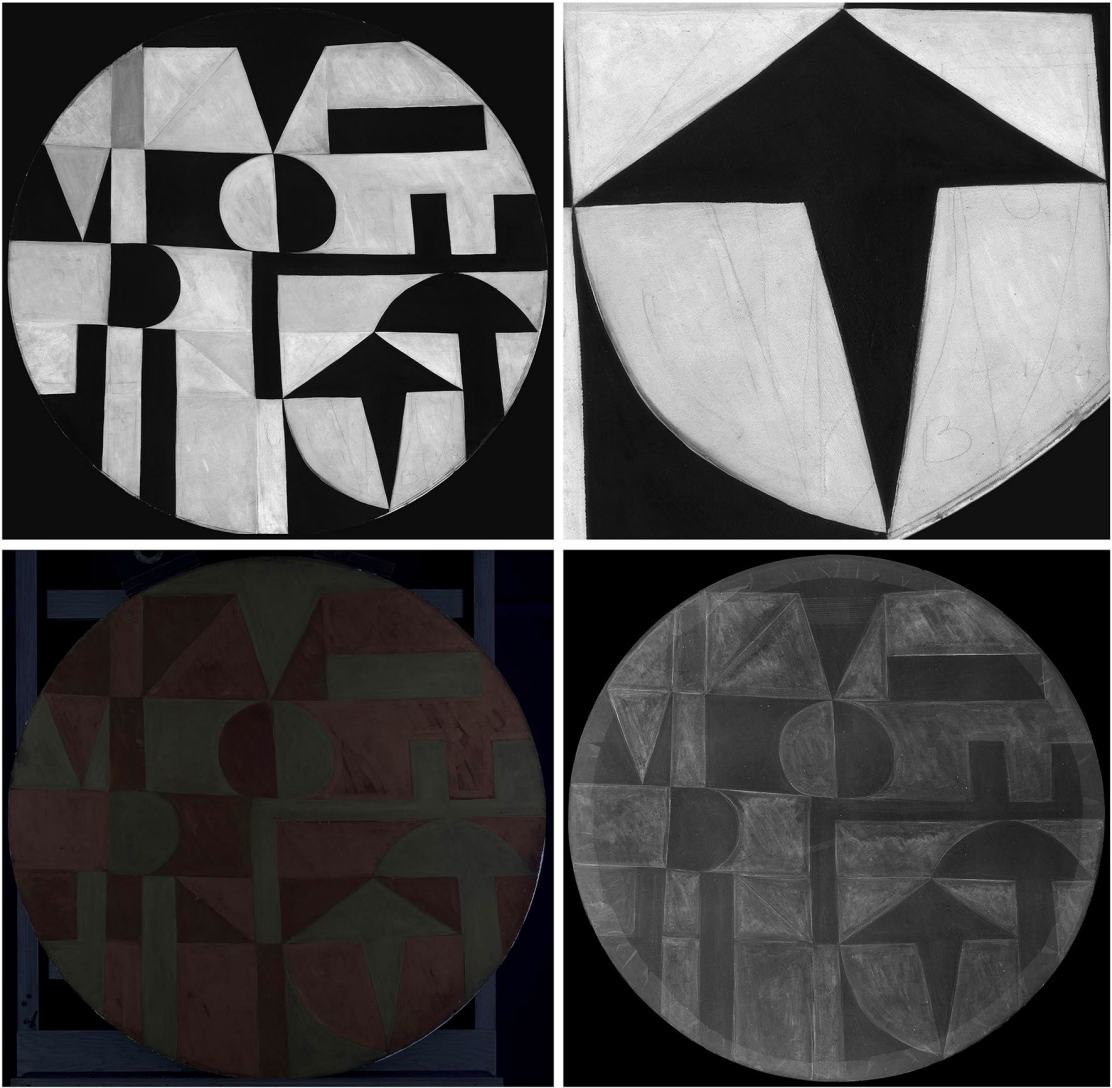
From top left to bottom right: IR reflectogram with detail, UV photograph, and X-radiograph of *Iberic* (1949)

X-radiography of *Iberic* did not uncover any compositional changes or underlying composition, yet it confirmed results from visual examination under normal light of a rather thinly painted surface (Fig. 10). In fact, Herrera’s painterly application is made more visible due to the radio-opacity of some of the pigments present in both the red and orange paints. Individual flat bristle brush marks reveal how the artist created her composition: first precisely following the linear outlines of each discrete geometric shape and then generally filling in the form with diagonal brushstrokes, starting at the upper left towards the bottom right, suggesting a right-handed application. Depending on the size of each individual geometric shape, Herrera appears to have used small or medium-size bristle brushes, whose imprints are clearly legible within the red and orange paint passages.

The artist’s methodical paint application pattern can be observed as well in the IR reflectogram (Fig. 10), which is quite revelatory about her drafting process for this work. Herrera loosely laid out her composition using fine pencil lines, sometimes going over those lines in a searching manner, and making numerous changes in the process. These fine lines, outlining the various geometric shapes within the composition, are mostly visible along and within red and orange sections, which, unlike the black paint, let the IR radiation penetrate the light-colored paint layers. All the compositional changes, including various rectangular, diagonal, and semi-circular pencil lines, show that the artist was working within the specific pictorial vocabulary she had set out for this composition, fine-tuning the placement of each shape in order to achieve a satisfactory visual balance. The IRR also indicates that Herrera was carefully planning the chromatic arrangement in the drafting stages of her composition as she made color notations, using single initial letters—“R” for “red”, “O” for “orange”, and “B” for “black”^4^—that are only visible in some of the red and orange shapes (Fig. 10). IRR images show that, within some individual shapes, these initials changed during the planning stages—for instance, from “B” to “R” or “O”. Interestingly, the final layer color sometimes does not match Herrera’s original pencil notations, indicating that she also made changes during the painting process. Also visible in the IRR is a thick pencil line traced along the circumference of the tondo panel, which is an additional evidence of Herrera’s early compositional planning. This distinctively drawn pencil line suggests that the artist had set the dimension of her composition early on, building it from a tondo she made out of a cut-out pebble paperboard, over which she stretched and glued a plain weave canvas prepared with a white ground layer. Finally, IRR images also reveal how the artist used a pencil to sign her work within the fresh red paint at the bottom of the composition. Herrera etched her last name with a fine pencil led while the paint was still wet, picking up the red paint in the process to expose the underlying ground layer, and leaving minor graphite traces in it, only detectable with IRR.

UV photography also unveiled both distinctive fluorescence and bristle brush application patterns, the latter seemingly unrelated to patterns visible in the IRR and X-radiograph discussed above (Fig. 10). Both features, however, suggest the presence of a locally applied surface coating, or a painting medium, presumably applied by the artist. The uneven application pattern, characterized by similar flat bristle brushes as the paint layer and by occasional drips running down vertically from one shape to another, all seem to support this hypothesis. According to optical microscopy images, most of the samples investigated did not clearly appear to include an organic coating at the top of the stratigraphy, with the exception of the following cross sections: S4, removed from a black area with severe medium reticulation at the work’s surface (Additional file 5: Fig. S5); S7, taken from a red field at the proper right edge of the painting; and S17, collected from a red area in the upper, proper left quadrant. These observations appear to confirm the presence of a varnish in selected areas of the painting.

**Fig. 11:**
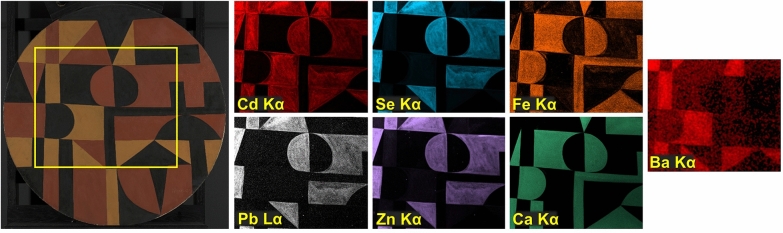
Elemental distribution maps of *Iberic* (1949) obtained by MA-XRF in an area of the painting indicated by a yellow rectangle: Cd Kα, Se Kα, Fe Kα, Ba Kα, Pb Lα, Zn Kα, and Ca Kα. The painting is also shown at left for comparison

Maps acquired by MA-XRF show the distribution of the main components in the red, orange, and black paints (Fig. 11). As in the case of *Iberia #25*, the red paint areas were found to contain Cd and Se, indicating that cadmium red is present. Pb- and Zn-based compounds were also observed in these regions. The fact that these Cd-, Pb- and Zn-rich materials are present in the same paint mixture can be corroborated by comparing the brushstrokes in the corresponding distribution maps. In the orange areas, Cd was detected as the main component, indicating that cadmium yellow is present. Relatively weaker signals for Zn and Se are observed in the orange areas, suggesting that these elements are either present in a cadmium red-containing paint in a layer below the top one and/or that some cadmium red-containing paint is mixed with cadmium yellow in the orange paint. As observed for *Iberia #25*, Ba is mostly present in the orange paint. Herrera’s painterly application of the red and orange fields, revealed by their relatively greater radio-opacity in X-radiography, is consistently reflected in the Cd, Se, Pb, and Zn distribution maps. In the black paints, Ca and Fe were identified. As discussed below, the presence of Ca in these areas is due to a bone or ivory black, and this element was also identified in the ground preparation of all samples analyzed. Relatively weaker Fe signals are detected in the maps of certain paint areas, particularly the red. However, Raman and SEM/EDS analysis did not identify any Fe-, Pb-, or Zn-containing materials in the cross sections examined, possibly due to issues related to the detection limits. Given the complexity of the paint layering and mixing of colors in this work, cross section analysis was once again necessary to determine the stratigraphy of the different paint passages and their composition with certainty.

All cross sections from *Iberic* were found to have a similar preparatory layer as that discussed for *Iberia #25*, composed of rutile, anhydrite, and talc. Two of these samples have a single paint layer over the ground preparation, and four display remnants of earlier paint applications right over the ground that appear to have been scraped off before new colored layers were applied. In detail, samples S7 and S10, removed from areas of red and orange paint, comprise only one paint layer, respectively composed of cadmium sulfoselenide pigments with red and orange hues. In regions of the painting where these two samples were taken, IRR revealed the artist’s pencil notes, “B” and “B” surrounded by “O”, respectively, possibly indicating that Herrera had originally planned on black painted fields; however, no evidence of additional paint applications was found in either case. On the other hand, cross section S5 (Additional file 6: Fig. S6), taken from a red field where a “B” pencil note was found with IRR, is comprised of two layers underneath the uppermost cadmium sulfoselenide and barite red paint: one is a mixture of a bone or ivory black with red cadmium sulfoselenide and barite particles, while the second consists of a cadmium sulfoselenide pigment with an orange hue. In the right portion of this sample, the latter is present in a thin paint layer; however, to the left, only scattered particles of this material are visible, suggesting that the artist may have scraped off the orange paint before applying first black followed by red paint layers. The same observation applies to cross sections S4, S6, and S9, removed from black (the first) and orange (the other two) paint areas. Sample S4 (Additional file 7: Fig. S7) is mostly composed of a mixture of a bone or ivory black with cadmium yellow, with a few Cd- and Se-containing red particles at the interface between the ground layer and the black paint. In cross section S6 (Additional file 8: Fig. S8), a cadmium sulfoselenide-containing layer with an orange hue is observed, under which remnants of an earlier application consisting of a cadmium sulfoselenide red paint with large bone or ivory black particles is present. In sample S9, traces of a black layer are seen below the main Cd-based orange paint layer. No indication of the desired color scheme by Herrera is visible in the orange field from which S6 was taken; however, “B” and “B” surrounded by “O” initials observed by IRR in areas corresponding to S5 and S9 (the latter removed from the edge of the same orange field as S10), red and orange respectively, hints at Herrera’s initial intention to apply black or orange paints.

Microscopic examination of the samples under UV illumination reveals, in some cases, the presence of abundant particles with a bright greenish-yellow fluorescence. These are visible, for example, in cross sections S5, S6, S7, S9, and S10 (Additional file 9: Fig. S9), in which they appear to be located within orange and red Cd-containing paint layers. SEM/EDS analysis showed that these luminescent particles are composed of cadmium yellow pigments that also contain Zn. In modern times, lighter yellow shades of cadmium pigments have been typically manufactured by partly substituting cadmium with zinc in the crystal structure, with up to 25% zinc (Cd_1-x_Zn_x_S) [14]. The luminescence properties of such cadmium zinc sulfide pigments have been reported in several studies [15–18]. As an alternative, physical mixtures of cadmium sulfide with white pigments and fillers, including barium sulfate, zinc sulfide, and zinc oxide, are also a possibility. Zinc oxide and zinc sulfide are also characterized by a luminescent behavior [19–23]. The fact that this luminescence was only observed for Cd-containing pigments in *Iberic*, but not for those in *Iberia #25*, points to the possible use of different types of paints by the artist in these two works. In addition, as observed for *Iberia #25*, in this case, too, Ba and Cd are detected in different particles, suggesting that Ba is most likely mixed in the cadmium paints, likely in the form of a filler.

As detailed in a previous publication by some of the authors [2], three samples of black (S1), orange (S2), and red paint (S3) were removed from Herrera’s *Iberic* for binding media analysis. The major component identified by Py-GC/MS in all these specimens was *n*-BMA. In addition, an oil-based binding medium was detected, as well as markers for Pinaceae resin, possibly indicating turpentine, commonly used as a solvent for early acrylics, or constituting an early enamel. Moreover, all three samples contain trace amounts of phthalic anhydride along with various phthalates, possibly present as plasticizers in the oil component. Traces or small amounts of styrene were also found. Interestingly, all samples were found to contain α-nicotine, indicating that the painting had been exposed to tobacco smoke.^5^

Five samples, namely S12, S14, S15, S16, and S18, are scrapings of surface coating from areas of the composition that appear to have an organic medium applied on top of the black, red, and orange paint. The coating in S12 was found to be composed primarily of oil modified to accelerate its drying (P/S = 1.5, A/P = 11.8), as well as *n*-BMA and phthalic acid. Traces of styrene and Pinaceae resin were also identified in this specimen. Analysis of samples S14-18, on the other hand, showed primarily *n*-BMA, with smaller amounts of methyl methacrylate, hexyl acrylate, dodecyl acrylate, and hexyldecyl acrylate, as well as relatively small quantities of drying oil (P/S = 2.0, A/P = 0.7). A trace of Pinaceae resin, but no styrene, was identified in this acrylic coating. The additional acrylic monomers do not necessarily reflect a different formulation, but rather the disparity in the proportion of the acrylic phase of the sample. This would appear to indicate that the black passages were selectively saturated by the application of a modified oil coating, while for the red and orange passages an acrylic coating was used. Because of the similarity in composition of the acrylic binder in the paints and the acrylic coating, it could be postulated that Herrera herself may have selectively applied these coatings. An additional sample (S13) was removed from a gummy residue found on the surface, which was identified as an adhesive from pressure sensitive tape derived from a 2-ethylhexyl acrylate polymer [24, 25].

### *Flights of Colors #16* (1949)

Three cross sections were removed from *Flights of Colors #16*, namely S2, S4, and S6, each from one of the three main hues in the painting—pink, orange, and light brown, respectively. All three samples show the same preparation as the two paintings discussed above and contain multiple paint layers. In cross section S2 (Additional file 10: Fig. S10), two layers are visible above the ground: a first light brown layer composed of a yellow ocher mixed with cadmium yellow and sparse, relatively large viridian particles; and a significantly thicker pink paint based on a combination of zinc white with an Al-, P-, and S-rich purpurin lake—as shown by SERS, the same colorant identified in *Iberia #25* (Fig. 7). Sample S4 (Additional file 11: Fig. S11) displays four paint layers, as follows: two distinct, but similar applications of an iron(III) oxide, containing abundant Fe-silicate particles; a thin, discontinuous layer composed of an Al-, P-, and S-rich organic lake; and a mixture of cadmium red, cadmium orange, and cadmium yellow with barite. In this case, the organic lake could not be identified by SERS likely due to the extreme thinness of the paint layer; however, visual examination by optical microscopy and EDS analysis suggests that it may be the same purpurin lake mentioned above. Cross section S6 (Additional file 12: Fig. S12) is characterized by an unusually complex stratigraphy compared to other specimens taken from this work, including at least seven layers with the following composition: cadmium red, iron(III) oxide, viridian, and a bone or ivory black; zinc white, a bone or ivory black, and traces of cobalt blue; zinc white, a bone or ivory black, and relatively larger amounts of cobalt blue; zinc white and Prussian blue; zinc white and Prussian blue, present in inverted relative proportions compared to the previous layer, thus explaining the more intense blue color; yellow ocher, mainly associated with Al silicates and Mg oxides; and zinc white with Fe- and Zn-rich particles.

As far as the binding media are concerned, two samples removed from pink (S1) and orange (S3) fields were found to be oil-based paints, possibly walnut or linseed mixed with poppy oil, or linseed oil with added heavy metal palmitates (P/S = 2.2, A/P = 0.6, and P/S = 1.8, A/P = 0.4, respectively). In the pink paint, unsaturated fatty acids, indicating incomplete drying, and Pinaceae resin are also present. The latter was also detected in the orange paint, along with traces of *n*-BMA. A sample of brown paint (S5) contains oil, possibly walnut or linseed mixed with poppy oil (P/S = 2.2, A/P = 1.0), and unsaturated fatty acids.

### *Early Dynasty* (1953)

For *Early Dynasty*, too, results confirmed the presence of a preparatory layer based on rutile, anhydrite, and talc. The paint stratigraphy was evaluated through visual examination and the analysis of five cross sections. Among these, S2 (Additional file 13: Fig. S13), S4, S5, and S6 were removed from areas of blue paint, while S9 (Additional file 14: Fig. S14) was taken from a red paint passage. The first three layers above the ground appear remarkably similar in all samples of blue paint, consisting mainly of cobalt blue (the first two) and Prussian blue (the latter), containing sparse particles of Mg-rich cerulean blue. While S2, S4, and S5 display only three layers, S6 contains one more, which was found to be based on ultramarine blue. Cross section S9 is characterized by two layers of cadmium sulfoselenide with a red hue.

Two sample scrapings, removed from an underlying blue paint layer (S1) and from the top blue paint (S3), are oil paints, possibly linseed, with an elevated azelaic acid amount, indicating accelerated drying (P/S = 1.3, A/P = 2.9, and P/S = 1.1, A/P = 3.7, respectively). Pinaceae resin was also identified in these samples. The red paint in sample S8 is also an oil paint, although it contains elevated levels of palmitic acid, presumably from the addition of heavy metal palmitates (P/S = 2.9, A/P = 0.6).

## Conclusions

Expanding on a preliminary study of Carmen Herrera’s pioneering use of solvent-based acrylic paints in post-war Europe [2], this article presents a second phase of research into the artist’s work aiming to ascertain the possible presence of other early acrylics in her Parisian artistic production (1948–1953) and to explore her painting techniques. This study focused on a selection of four works, namely *Iberia #25* (1948), *Iberic* (1949), *Flights of Colors #16* (1949), and *Early Dynasty* (1953). Questions investigated included the identification of binding media, as well as pigments, colorants, and extenders in the ground and paint layers. Careful inspection of the paint stratigraphy was also carried out to uncover unknown details of Herrera’s studio practice.

As in the authors’ previous study [2], results confirmed the use of both oil and solvent-based acrylic paints in this group of works, revealing the first-known occurrence of acrylic binders in Herrera’s *Iberia #25*, dated to 1948. Particularly striking is the artist’s use of both oil and early solvent-based acrylics in the earlier paintings (*Iberia #25* and *Iberic*), but only oil in the later works (*Flights of Colors #16* and *Early Dynasty*). This finding appears to confirm the availability of solvent-based acrylic paints to Herrera in Paris as early as 1948. It also allows one to pose the question as to whether her use of different paint binders for specific colored passages in the early paintings was a thoughtful and intentional selection, or just a case of availability. From the color notations and color changes that we observed in *Iberia #25* and *Iberic*, it is clear that Herrera was very deliberate about her polychromy choices. Similarly, the surface sheen or lack thereof of each color field was probably also important to her, which would explain the presence of selective coating in *Iberic*. While the working properties of Herrera’s historic paints is not known, solvent-based acrylic paints were derived initially to mimic the solubility of oil paints, thinnable in turpentine, while decreasing the drying time. Working in the living room of her Paris apartment, faster drying paints would have been attractive to Herrera. In her early works, many of the oil paints exhibited high azelaic acid contents, indicating that their drying time was accelerated. These results could indicate that the working properties of solvent-based acrylics and oils with elevated azelaic acid were similar enough that Herrera might not have made a distinction in using them, but rather employed paints that best suited her compositional color scheme. The artist’s exclusive use of oil paints in the later works would also seem to corroborate this hypothesis.

In all cases, the ground layer consists of a mixture of titanium white in the form of rutile, anhydrite, and talc. The color palette was found to be based on a relatively broad array of pigments, including bone or ivory black, Prussian blue, cobalt blue, cerulean blue, ultramarine blue, yellow and red ochers, cadmium-based pigments, a purpurin lake, viridian, and zinc white. Microscopic examination of cross sections revealed how Herrera’s creative process typically developed through subsequent compositions. In most instances, multiple layers are indeed present below the thick surface paint in monochromatic areas, sometimes of a similar color as the uppermost paint, but with different chemical composition. In some cases, remnants of earlier paint layers, which appeared to have been scraped off before new ones were applied, were observed right above the ground preparation. This frequently encountered practice of overpainting may be indicative of the artist changing her mind and altering the original color scheme, although it is also possible that, in some cases, she intentionally overlapped different colors to obtain particular hues and visual effects. Additionally, upon incorporation into The Met’s collection, *Iberic*—featured in the authors’ former study—underwent a more in-depth documentation along with *Iberia #25*, characterized by analogous geometric design and chromatic arrangement. Interestingly, investigation of these two works uncovered similarities in the choice of materials and, in the case of *Iberic*, exposed pencil lines and notes below the paint layers that are indicative of the intended geometric and chromatic scheme in specific areas. These findings clearly show how Herrera has carefully planned her compositions starting from the early phases of the creative process, relying at the same time on later adjustments and alterations.

Besides corroborating a major update in the current scholarship regarding the availability and use of solvent-based acrylic artists’ paints in post-war Europe, this research provides new insights into Herrera’s materials and studio practice. In addition, the results of this scientific study assisted the development of a suitable treatment plan for *Iberic* in preparation for display in The Met’s galleries as part of the 150th anniversary exhibition *Making The Met, 1870–2020*.

## Supplementary Information

**Additional file 1: Figure S1.** From left to right, sampling sites for *Iberia #25* (1948), *Iberic* (1949), *Flights of Colors #16* (1949), and *Early Dynasty* (1953).

**Additional file 2: Figure S2.** Combined elemental distribution maps for Cd Kα (red) and Se Kα (blue) obtained by MA-XRF for *Iberia #25* (1948, left) and for a selected area in *Iberic* (1949, right).

**Additional file 3: Figure S3.** Elemental distribution maps of *Iberia #25* (1948) obtained by MA-XRF: Ba Kα, Ba Lα, and composite Ba Lα (blue) and Ti Kα (red). The Ba Kα map shows scattering from the stretcher. The painting is also shown at bottom right for comparison.

**Additional file 4: Figure S4.** Left, representative Raman spectra of some of the pigments identified in cross sections S3, S6, and S8 from *Iberia #25* (1948), including **a** carbon-based black, **b** Prussian blue, and **c**–**f** cadmium yellow from yellow, orange, brown, and red areas, respectively. Right, EDS spectrum with intense Ca and P peaks, indicating bone or ivory black.

**Additional file 5: Figure S5.** Reticulation pattern of a coating layer visible on top of the black field of *Iberic* (1949) near site where cross section S4 was removed.

**Additional file 6: Figure S6.** Left, polarized light and UV light microphotographs of cross section S5 from *Iberic* (1949), with BSE image of a portion of the sample indicated by a yellow rectangle. Right, EDS elemental maps of Cd Lα, Se Kα, S Kα, Ca Kα, P Kα, Mg Kα, Si Kα, and Ti Kα.

**Additional file 7: Figure S7.** Left, polarized light and UV light microphotographs of cross section S4 from *Iberic* (1949), with BSE image of a portion of the sample indicated by a yellow rectangle. Right, EDS elemental maps of Cd Lα, S Kα, Fe Kα, Ca Kα, Al Kα, P Kα, Mg Kα, Si Kα, and Ti Kα.

**Additional file 8: Figure S8.** Left, polarized light and UV light microphotographs of cross section S6 from *Iberic* (1949), with BSE image of a portion of the sample indicated by a yellow rectangle. Right, EDS elemental maps of Cd Lα, Se Kα, S Kα, Ca Kα, Ba Lα, P Kα, Mg Kα, Si Kα, and Ti Kα.

**Additional file 9: Figure S9.** UV light microphotographs of cross sections S5, S6, S7, S9, and S10 from *Iberic* (1949), displaying particles with a bright greenish-yellow fluorescence.

**Additional file 10: Figure S10.** Left, polarized light and UV light microphotographs of cross section S2 from *Flights of Colors #16* (1949), with BSE image of a portion of the sample indicated by a yellow rectangle. Right, EDS elemental maps of Cd Lα, S Kα, Fe Kα, Zn Kα, Al Kα, P Kα, Ca Kα, Si Kα, and Cr Kα.

**Additional file 11: Figure S11.** Left, polarized light and UV light microphotographs of cross section S4 from *Flights of Colors #16* (1949), with BSE image of a portion of the sample indicated by a yellow rectangle. Right, EDS elemental maps of Cd Lα, Se Kα, S Kα, Ca Kα, Al Kα, P Kα, Ba Lα, Fe Kα, and Cr Kα.

**Additional file 12: Figure S12.** Left, polarized light and UV light microphotographs of cross section S6 from *Flights of Colors #16* (1949), with BSE image of a portion of the sample indicated by a yellow rectangle. Right, EDS elemental maps of Cd Lα, Cr Kα, S Kα, Ca Kα, P Kα, Zn Kα, Co Kα, Al Kα, Si Kα, and Fe Kα.

**Additional file 13: Figure S13.** Left, polarized light and UV light microphotographs of cross section S2 from *Early Dynasty* (1953), with BSE image of a portion of the sample indicated by a yellow rectangle. Right, EDS elemental maps of Fe Kα, Co Kα, S Kα, Ca Kα, Al Kα, Mg Kα, Si Kα, and Ti Kα.

**Additional file 14: Figure S14.** Left, polarized light and UV light microphotographs of cross section S9 from *Early Dynasty* (1953), with BSE image of a portion of the sample indicated by a yellow rectangle. Right, EDS elemental maps of Cd Lα, Se Kα, S Kα, Ca Kα, Al Kα, P Kα, Zn Kα, Si Kα, and Ti Kα.

## Data Availability

All data generated during this study are either included in this published article or available from the corresponding author upon reasonable request.

## Abbreviations

IRR: Infrared reflectography
MA-XRF: Macro-X-ray fluorescence spectroscopy
SEM/EDS: Scanning electron microscopy with energy-dispersive X-ray spectroscopy
BSE: Back-scattered electron
SERS: Surface-enhanced Raman spectroscopy
FTIR: Fourier-transform infrared spectroscopy
Py-GC/MS: Pyrolysis-gas chromatography/mass spectrometry

## Footnotes

1 Email exchange between Dana Miller and Isabelle Duvernois, September 9th, 2021.

2 See footnote 27 in reference 1.

3 Personal communication with Tony Bechara, July 24th, 2019.

4 Interestingly, Herrera, whose first language is Spanish, chose to write such notations in English, her adopted language and the one she would have spoken with her husband while both were living in Paris. In French, “R”, “O”, and “B” initials could also stand for “rouge”, “orange”, and “bleu”. However, while Prussian blue layers were identified in a few cases under the black paint in *Iberia #25*, these were never observed in *Iberic*, making the hypothesis of French initials unlikely.

5 Herrera and her husband Jesse Loewenthal did have many friends and often gathered at their apartment at 5 Rue Campagne Première in Paris, which also functioned as a studio for Carmen. As nearly everyone smoked in France at that time, it is very likely that is where the nicotine exposure took place.
