# Supplementary figures and images for: A pioneer of acrylic painting: new insights into Carmen Herrera’s studio practice
Source: Herit Sci. 2021 Oct 14;9(1):131. doi: 10.1186/s40494-021-00603-3 (PMC8515334; doi:10.1186/s40494-021-00603-3)

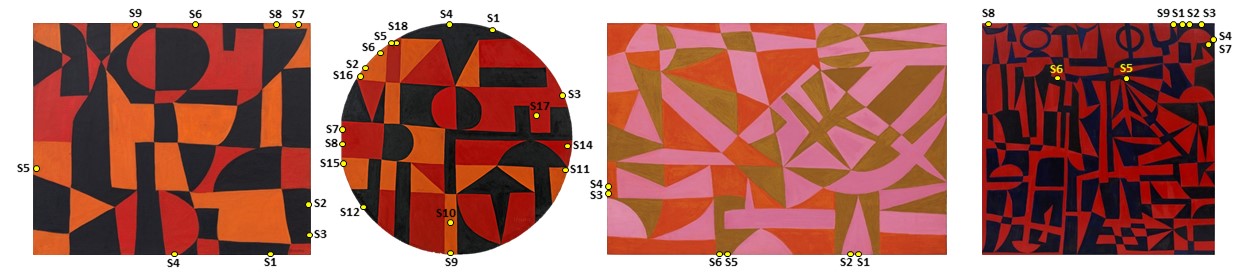

Supplement: Supplementary file 1 — Additional file 1: Figure S1. From left to right, sampling sites for Iberia #25 (1948), Iberic (1949), Flights of Colors #16 (1949), and Early Dynasty (1953). [file 40494_2021_603_MOESM1_ESM.jpg]

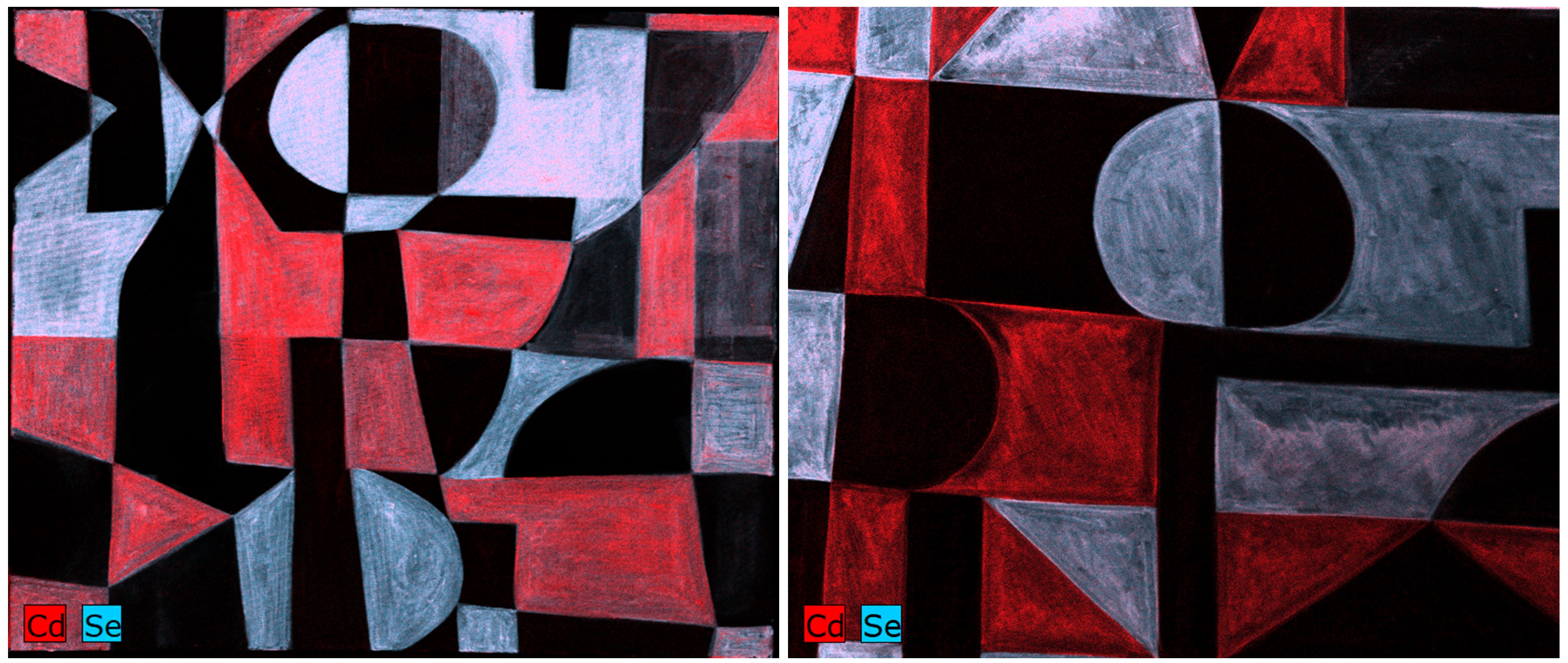

Supplement: Supplementary file 2 — Additional file 2: Figure S2. Combined elemental distribution maps for Cd Kα (red) and Se Kα (blue) obtained by MA-XRF for Iberia #25 (1948, left) and for a selected area in Iberic (1949, right). [file 40494_2021_603_MOESM2_ESM.jpg]

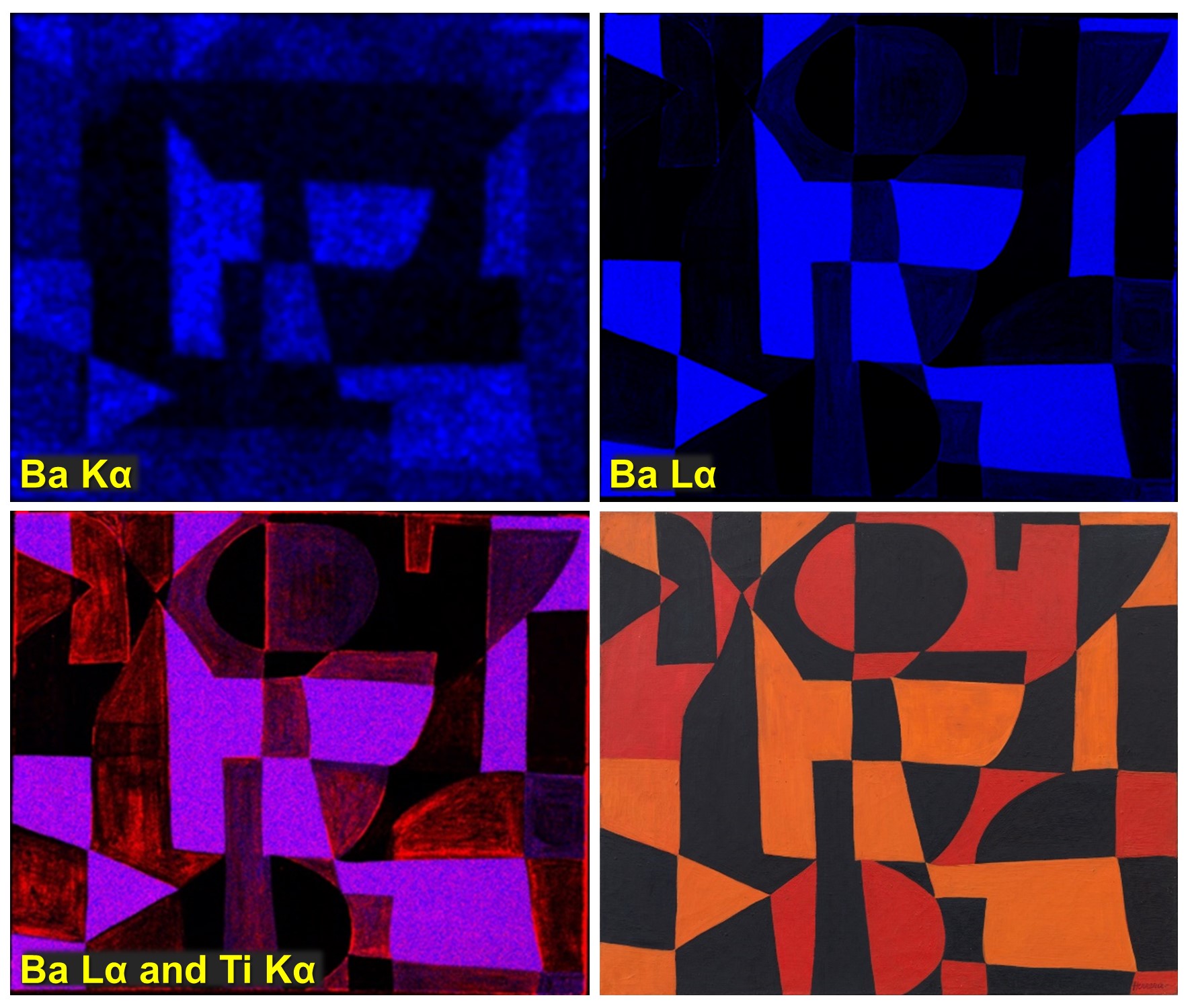

Supplement: Supplementary file 3 — Additional file 3: Figure S3. Elemental distribution maps of Iberia #25 (1948) obtained by MA-XRF: Ba Kα, Ba Lα, and composite Ba Lα (blue) and Ti Kα (red). The Ba Kα map shows scattering from the stretcher. The painting is also shown at bottom right for comparison. [file 40494_2021_603_MOESM3_ESM.jpg]

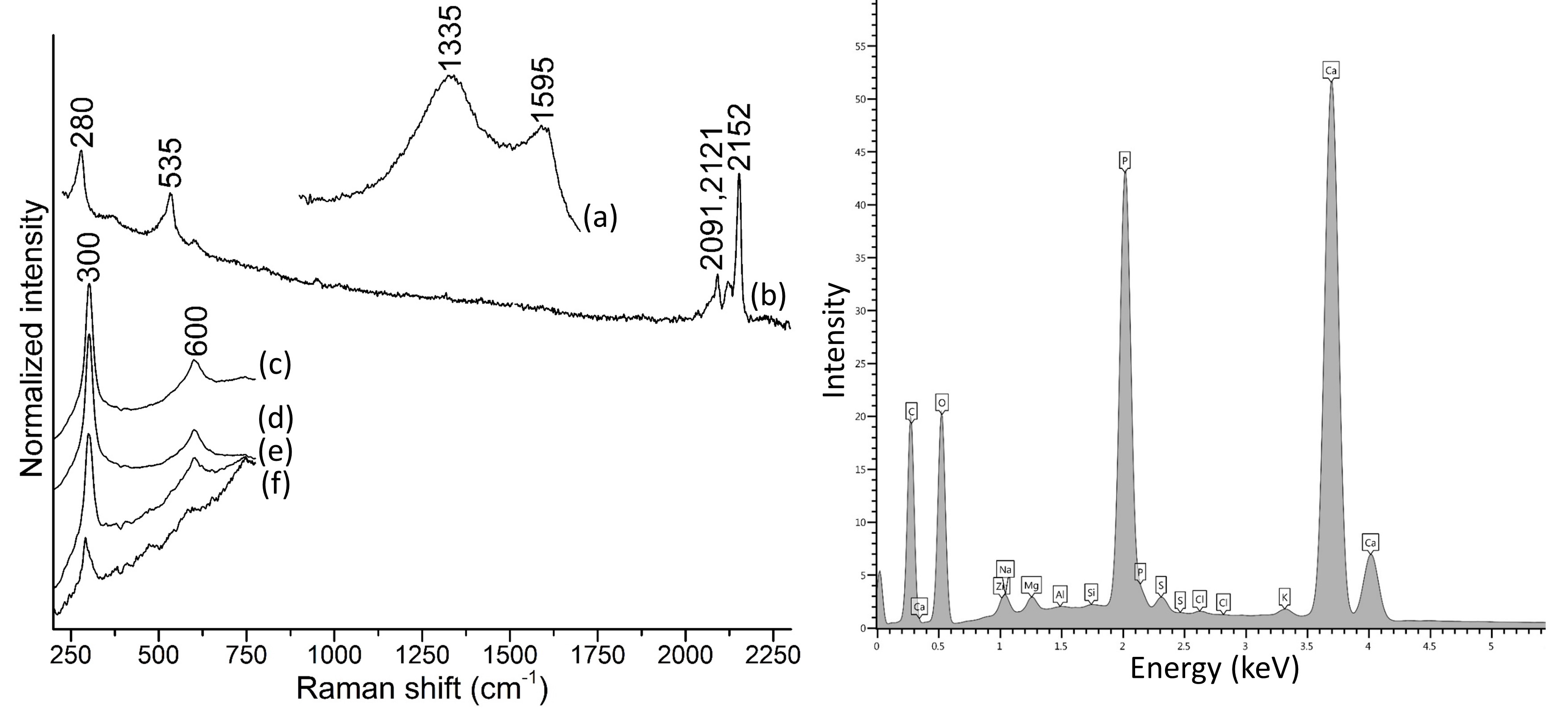

Supplement: Supplementary file 4 — Additional file 4: Figure S4. Left, representative Raman spectra of some of the pigments identified in cross sections S3, S6, and S8 from Iberia #25 (1948), including a carbon-based black, b Prussian blue, and c–f cadmium yellow from yellow, orange, brown, and red areas, respectively. Right, EDS spectrum with intense Ca and P peaks, indicating bone or ivory black. [file 40494_2021_603_MOESM4_ESM.jpg]

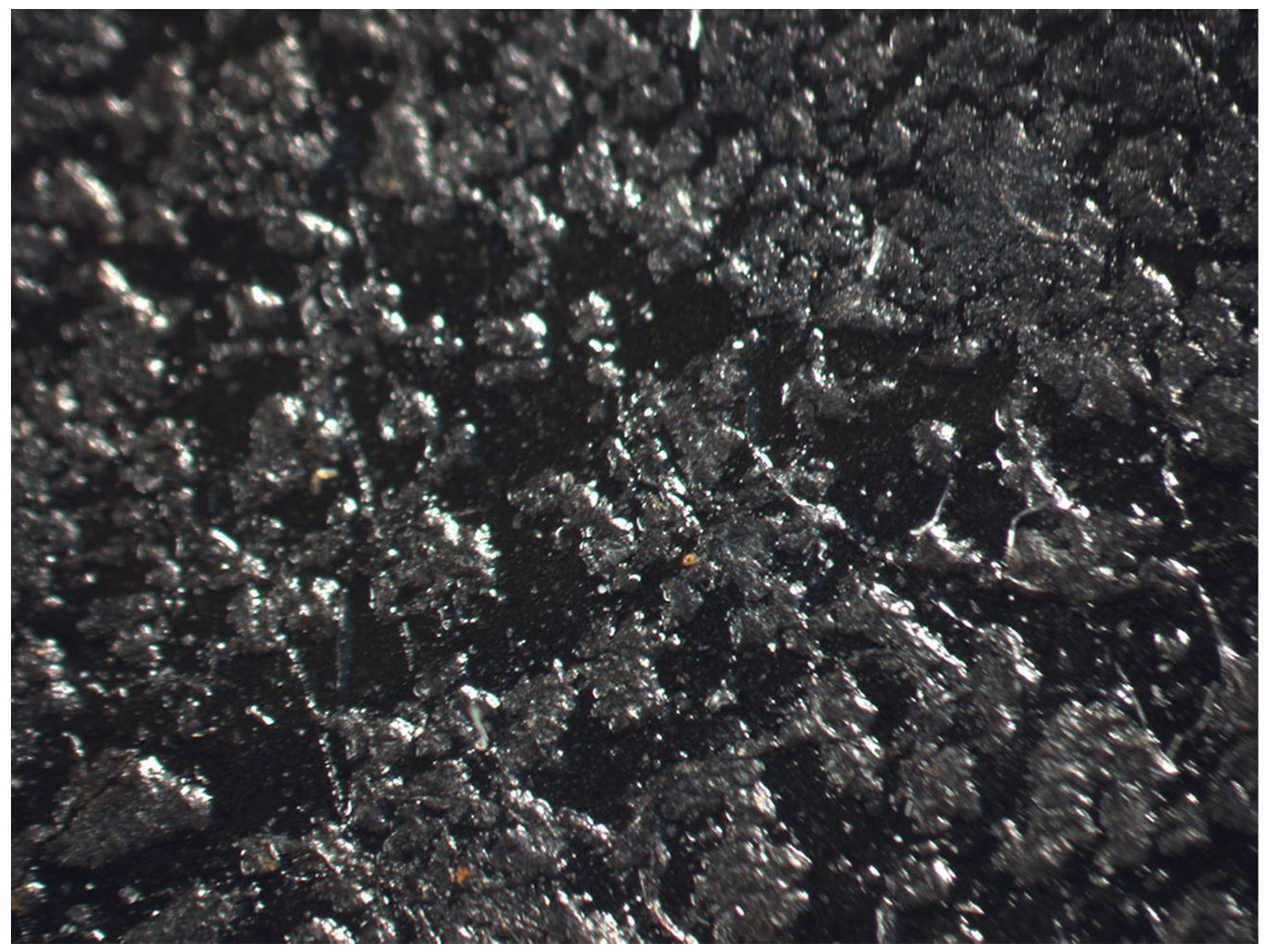

Supplement: Supplementary file 5 — Additional file 5: Figure S5. Reticulation pattern of a coating layer visible on top of the black field of Iberic (1949) near site where cross section S4 was removed. [file 40494_2021_603_MOESM5_ESM.jpg]

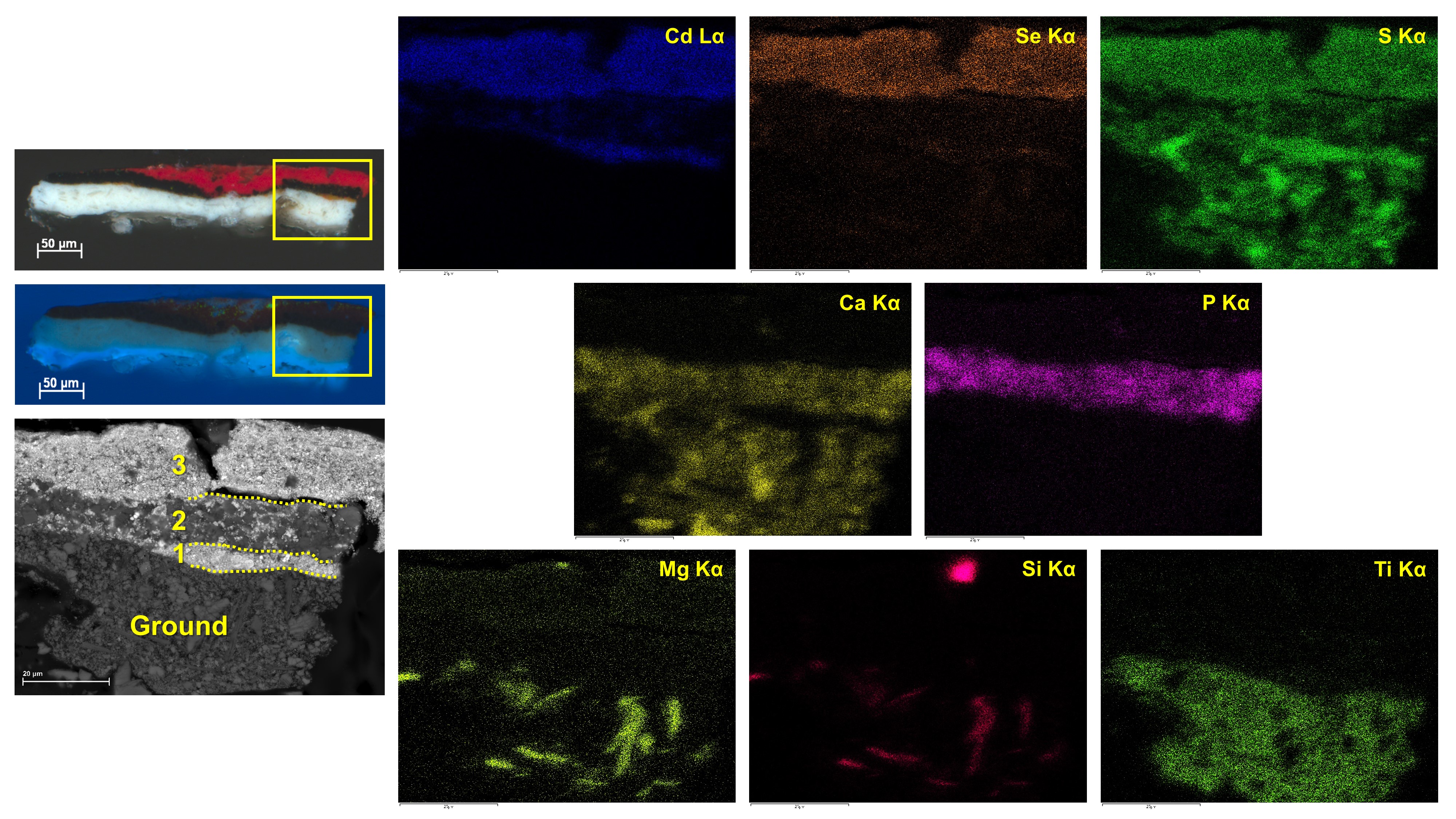

Supplement: Supplementary file 6 — Additional file 6: Figure S6. Left, polarized light and UV light microphotographs of cross section S5 from Iberic (1949), with BSE image of a portion of the sample indicated by a yellow rectangle. Right, EDS elemental maps of Cd Lα, Se Kα, S Kα, Ca Kα, P Kα, Mg Kα, Si Kα, and Ti Kα. [file 40494_2021_603_MOESM6_ESM.jpg]

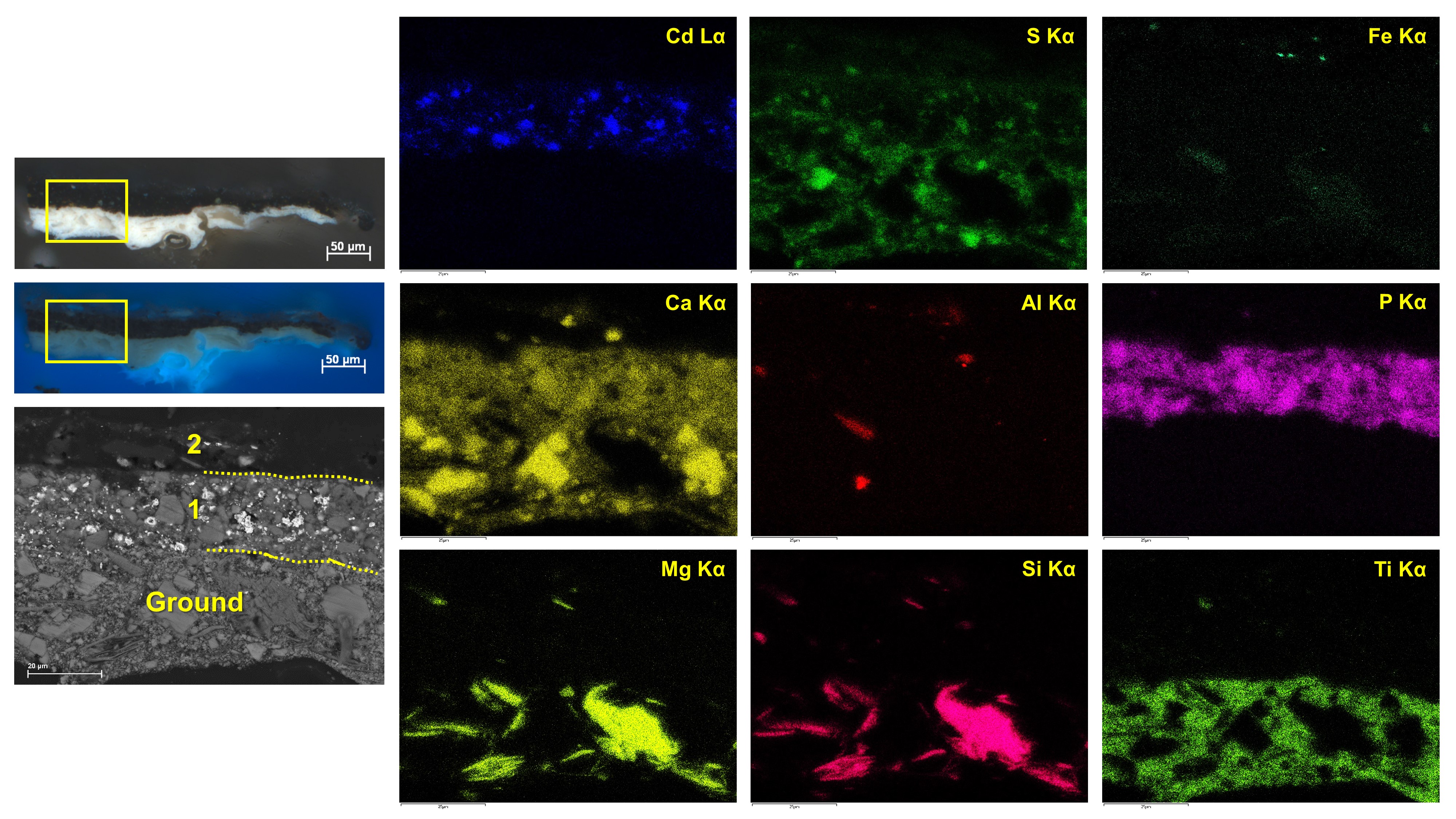

Supplement: Supplementary file 7 — Additional file 7: Figure S7. Left, polarized light and UV light microphotographs of cross section S4 from Iberic (1949), with BSE image of a portion of the sample indicated by a yellow rectangle. Right, EDS elemental maps of Cd Lα, S Kα, Fe Kα, Ca Kα, Al Kα, P Kα, Mg Kα, Si Kα, and Ti Kα. [file 40494_2021_603_MOESM7_ESM.jpg]

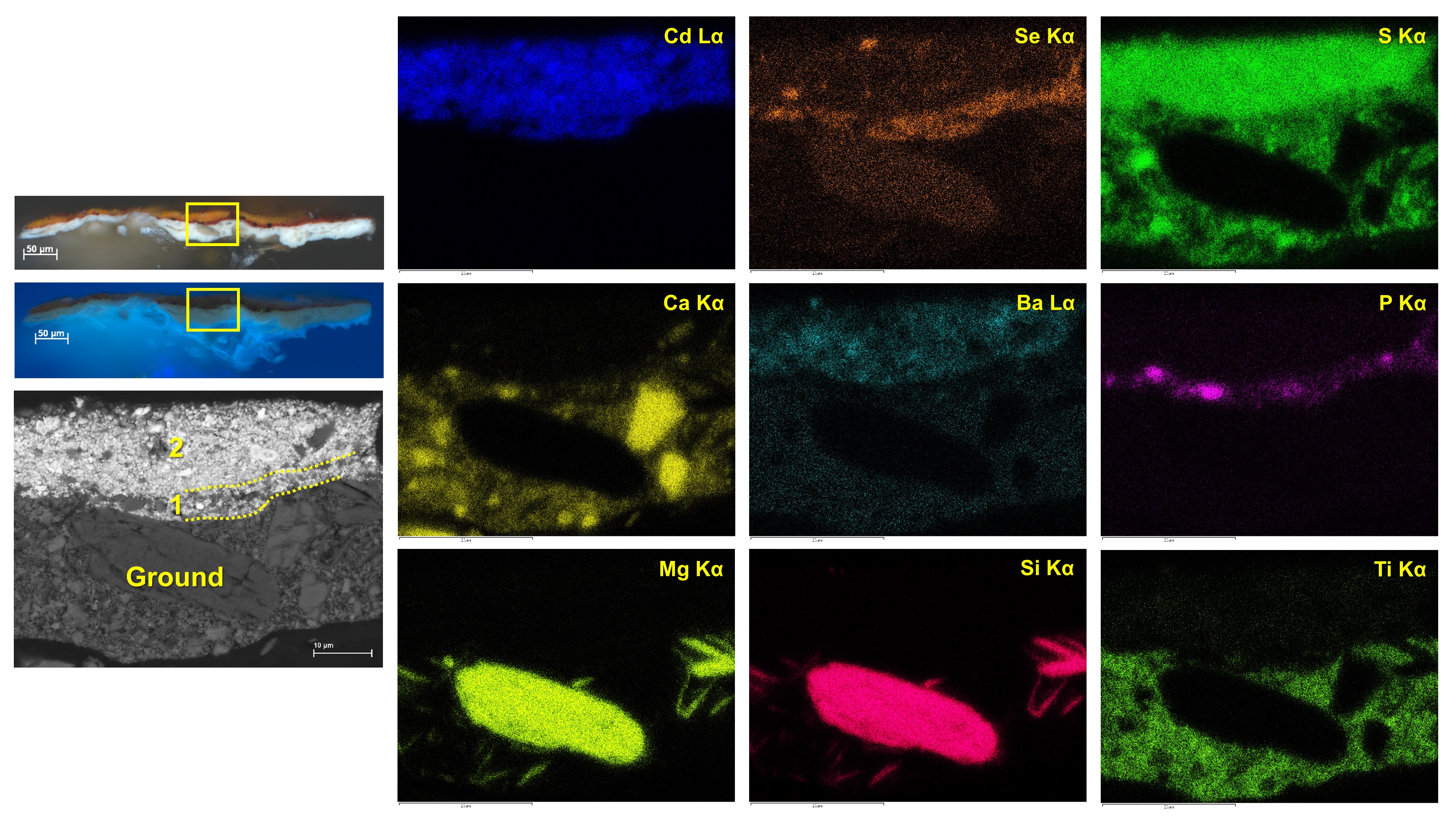

Supplement: Supplementary file 8 — Additional file 8: Figure S8. Left, polarized light and UV light microphotographs of cross section S6 from Iberic (1949), with BSE image of a portion of the sample indicated by a yellow rectangle. Right, EDS elemental maps of Cd Lα, Se Kα, S Kα, Ca Kα, Ba Lα, P Kα, Mg Kα, Si Kα, and Ti Kα. [file 40494_2021_603_MOESM8_ESM.jpg]

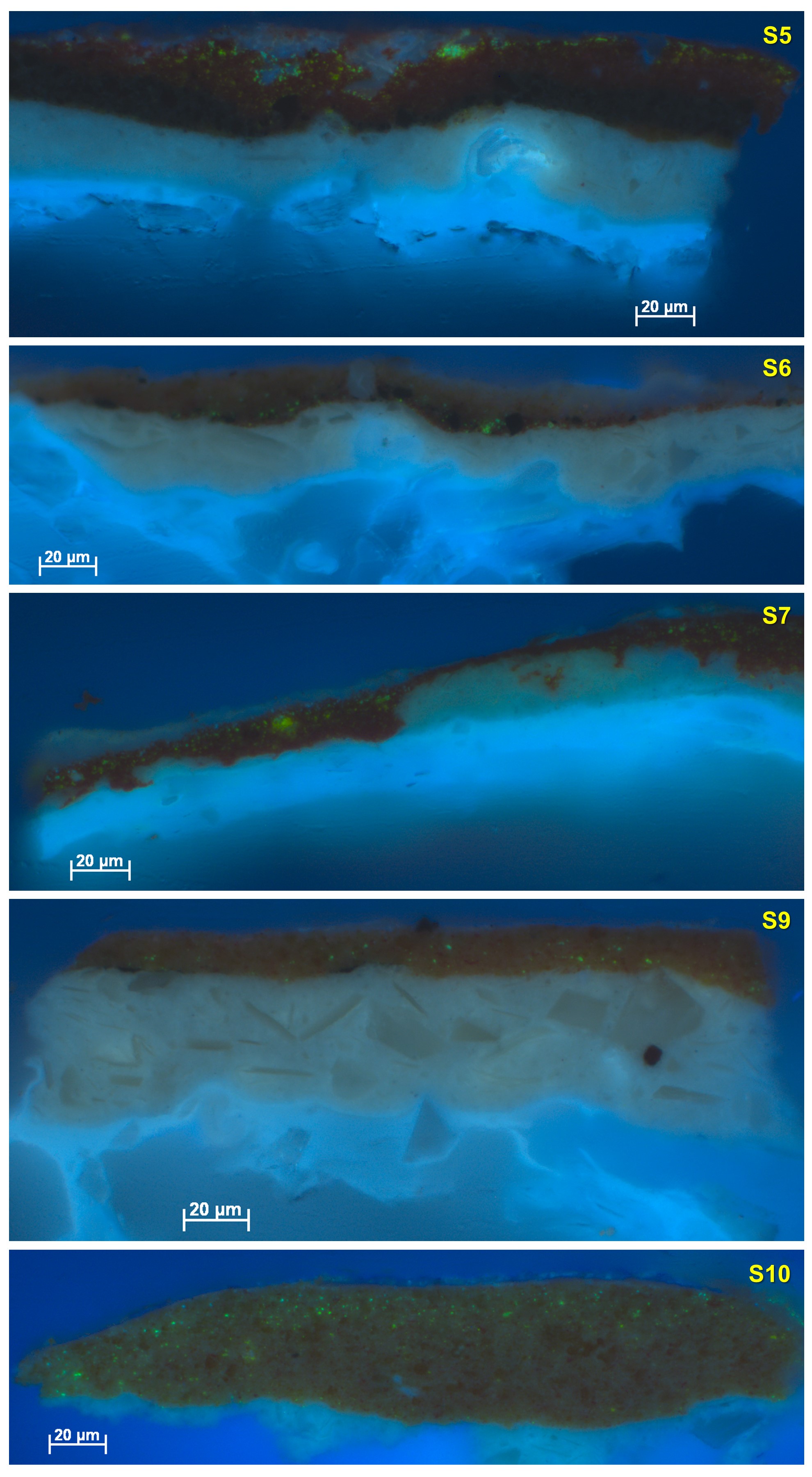

Supplement: Supplementary file 9 — Additional file 9: Figure S9. UV light microphotographs of cross sections S5, S6, S7, S9, and S10 from Iberic (1949), displaying particles with a bright greenish-yellow fluorescence. [file 40494_2021_603_MOESM9_ESM.jpg]

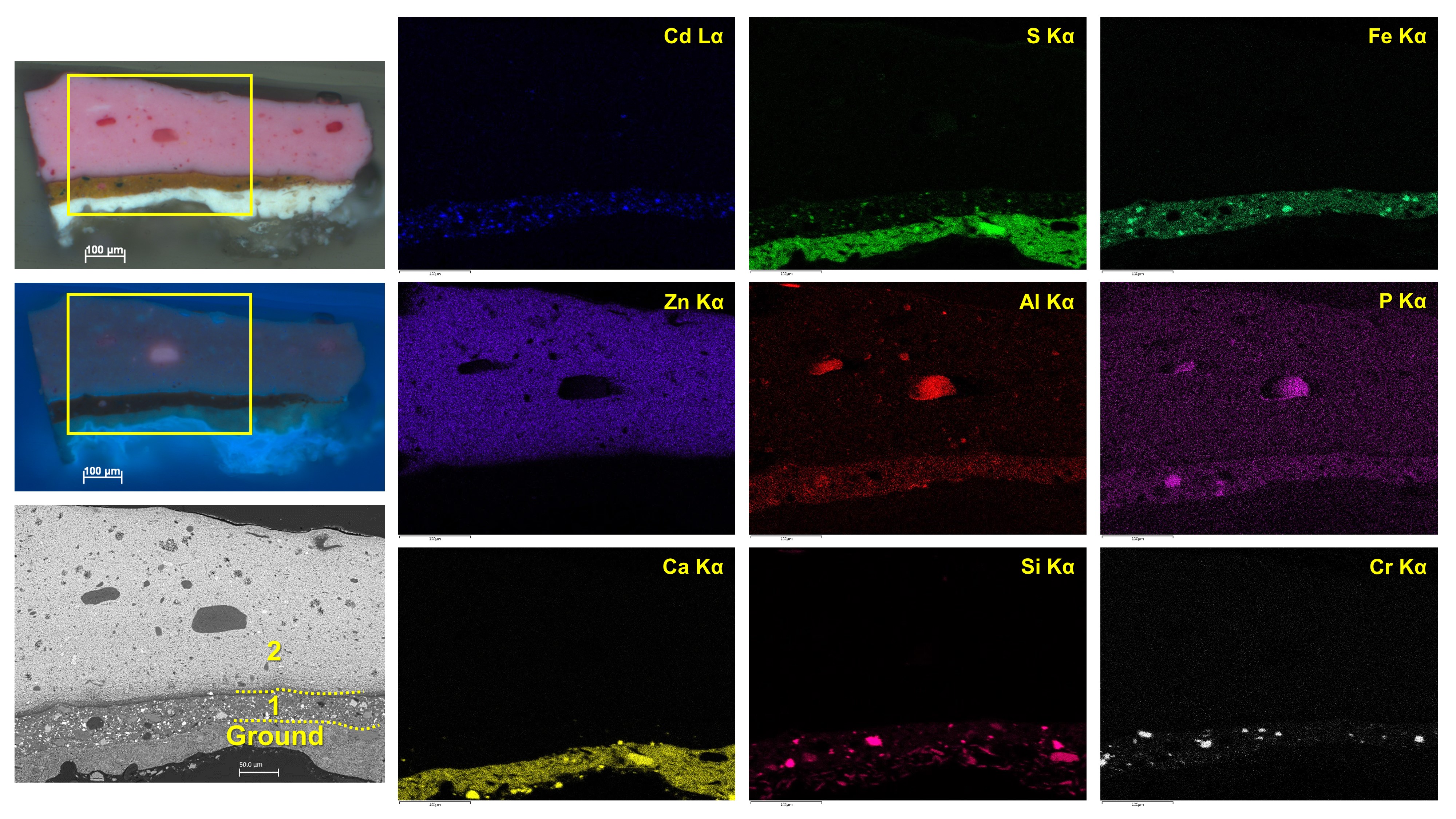

Supplement: Supplementary file 10 — Additional file 10: Figure S10. Left, polarized light and UV light microphotographs of cross section S2 from Flights of Colors #16 (1949), with BSE image of a portion of the sample indicated by a yellow rectangle. Right, EDS elemental maps of Cd Lα, S Kα, Fe Kα, Zn Kα, Al Kα, P Kα, Ca Kα, Si Kα, and Cr Kα. [file 40494_2021_603_MOESM10_ESM.jpg]

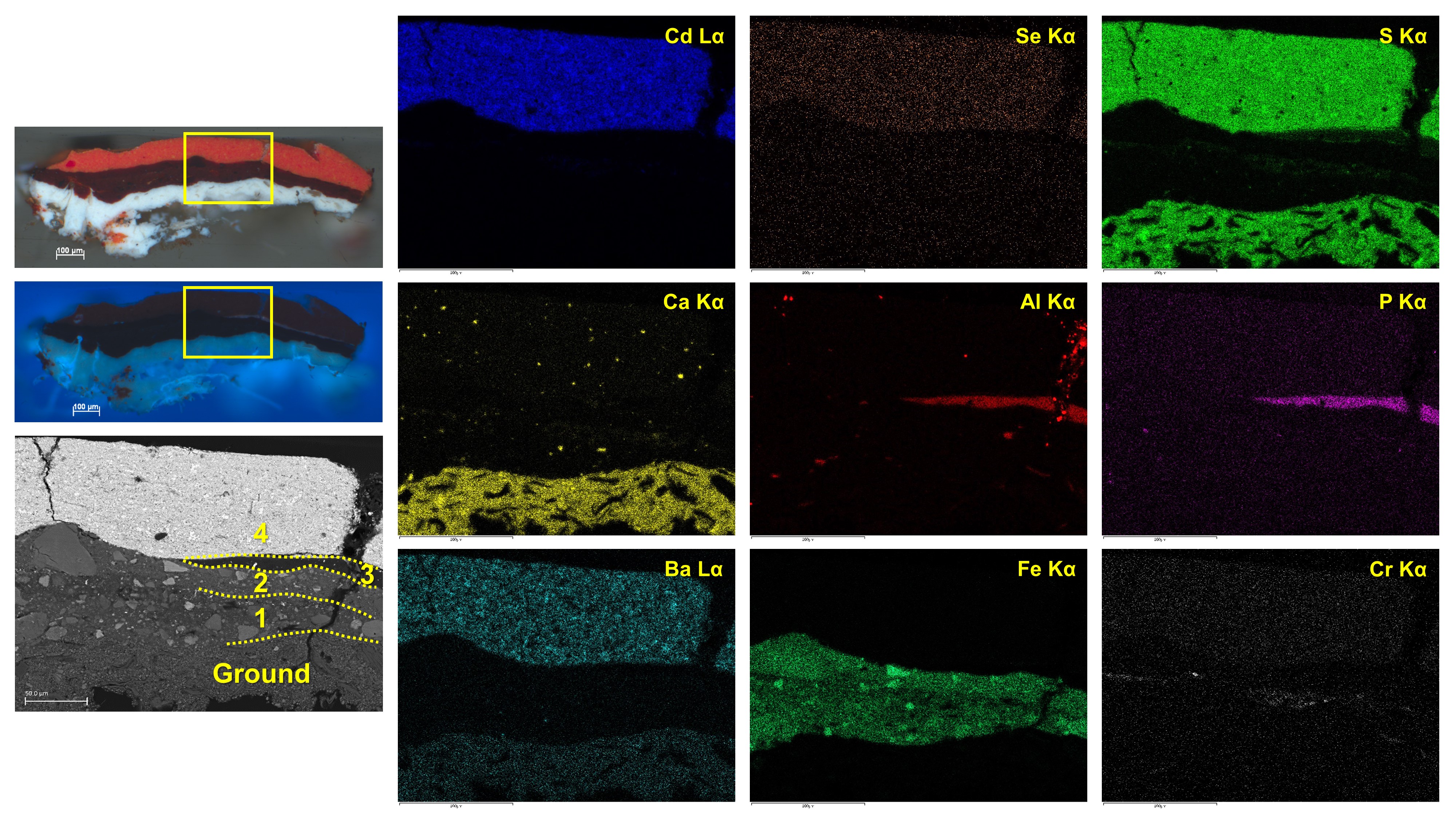

Supplement: Supplementary file 11 — Additional file 11: Figure S11. Left, polarized light and UV light microphotographs of cross section S4 from Flights of Colors #16 (1949), with BSE image of a portion of the sample indicated by a yellow rectangle. Right, EDS elemental maps of Cd Lα, Se Kα, S Kα, Ca Kα, Al Kα, P Kα, Ba Lα, Fe Kα, and Cr Kα. [file 40494_2021_603_MOESM11_ESM.jpg]

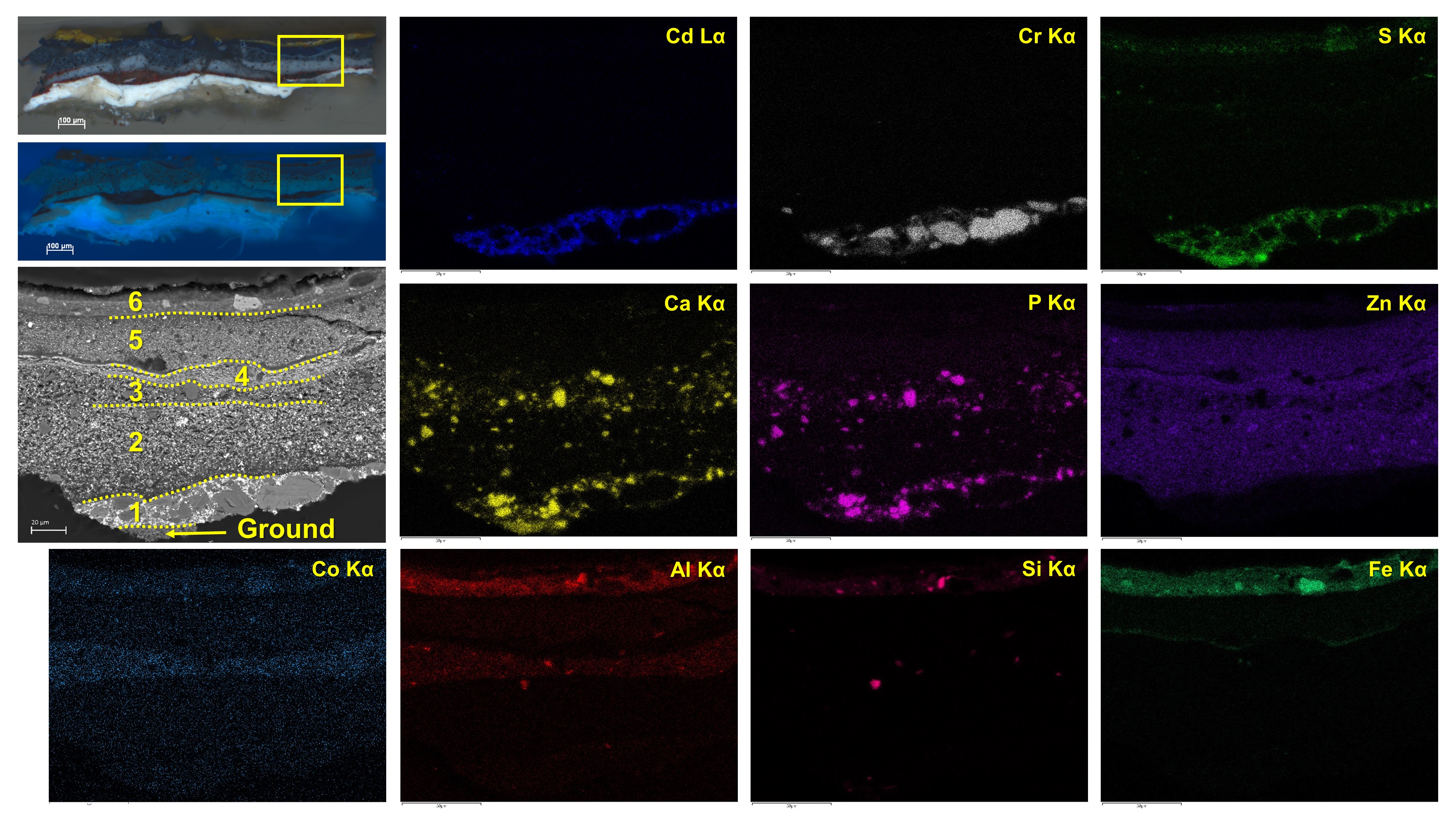

Supplement: Supplementary file 12 — Additional file 12: Figure S12. Left, polarized light and UV light microphotographs of cross section S6 from Flights of Colors #16 (1949), with BSE image of a portion of the sample indicated by a yellow rectangle. Right, EDS elemental maps of Cd Lα, Cr Kα, S Kα, Ca Kα, P Kα, Zn Kα, Co Kα, Al Kα, Si Kα, and Fe Kα. [file 40494_2021_603_MOESM12_ESM.jpg]

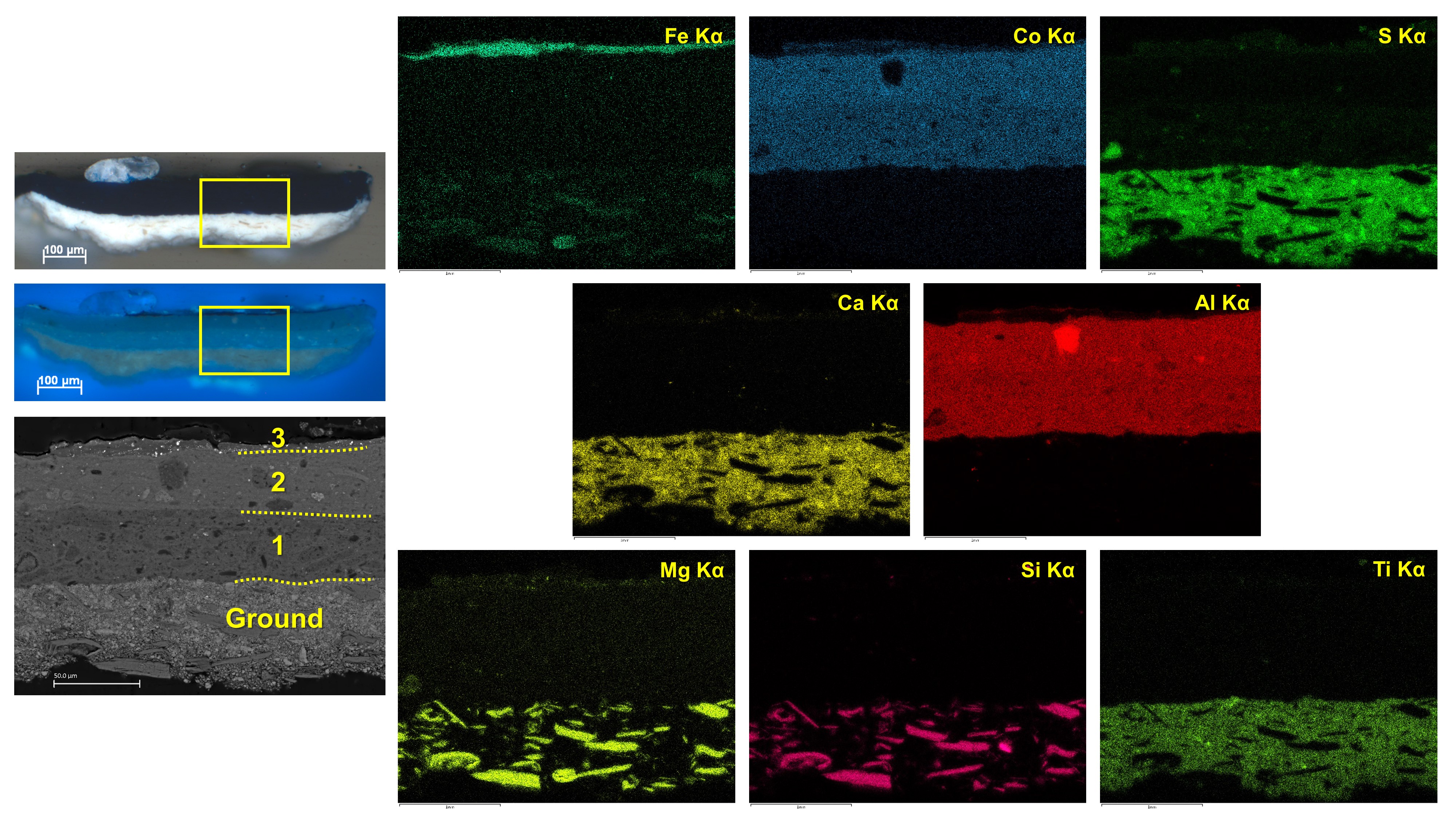

Supplement: Supplementary file 13 — Additional file 13: Figure S13. Left, polarized light and UV light microphotographs of cross section S2 from Early Dynasty (1953), with BSE image of a portion of the sample indicated by a yellow rectangle. Right, EDS elemental maps of Fe Kα, Co Kα, S Kα, Ca Kα, Al Kα, Mg Kα, Si Kα, and Ti Kα. [file 40494_2021_603_MOESM13_ESM.jpg]

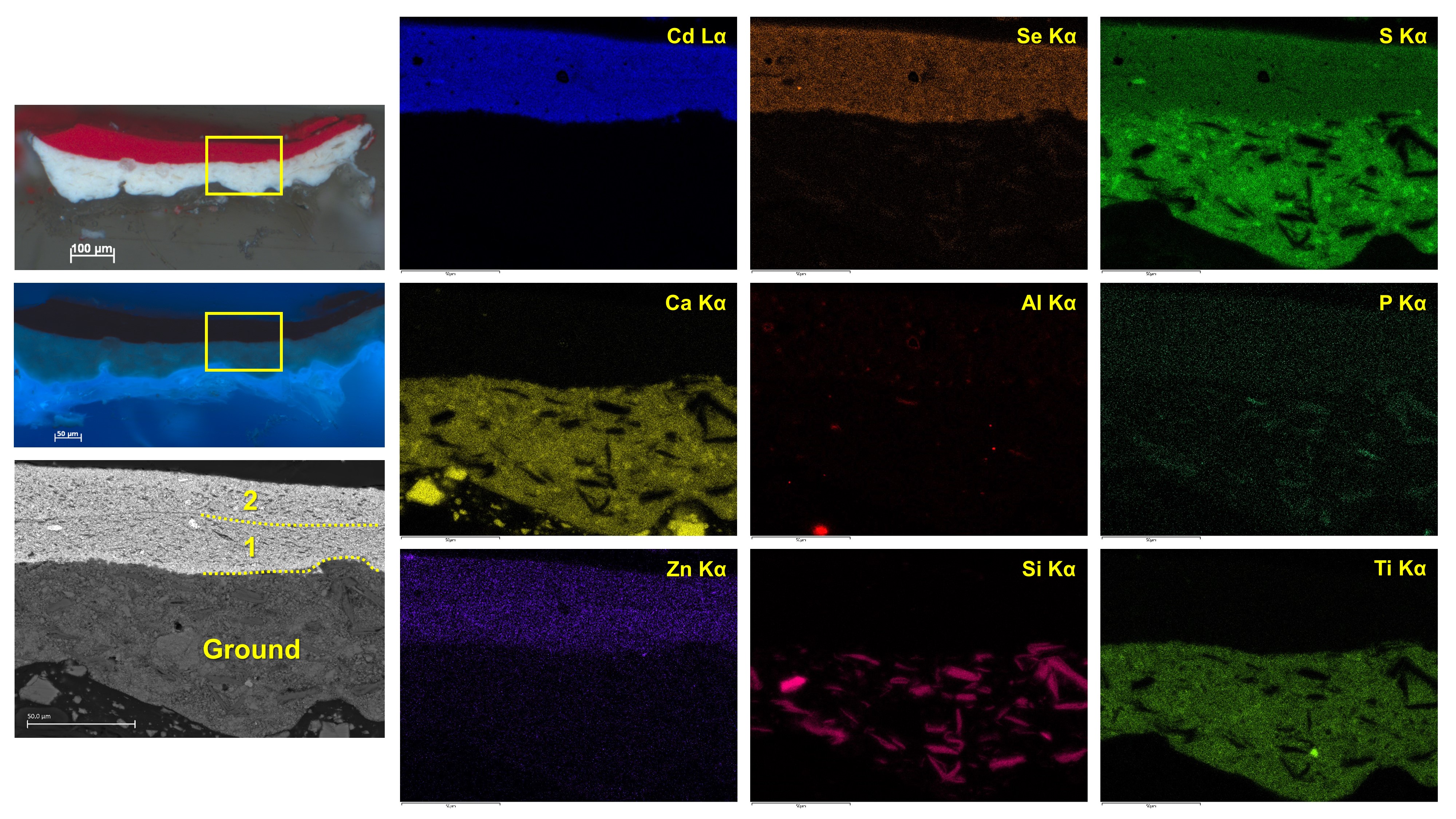

Supplement: Supplementary file 14 — Additional file 14: Figure S14. Left, polarized light and UV light microphotographs of cross section S9 from Early Dynasty (1953), with BSE image of a portion of the sample indicated by a yellow rectangle. Right, EDS elemental maps of Cd Lα, Se Kα, S Kα, Ca Kα, Al Kα, P Kα, Zn Kα, Si Kα, and Ti Kα. [file 40494_2021_603_MOESM14_ESM.jpg]
